# Development and External Validation of Machine Learning Models for Ionized Calcium Estimation in Hypoalbuminemia

**DOI:** 10.3390/diagnostics16203381

**Published:** 2026-10-17

**Authors:** Levent Deniz, Tugay Solar, Ipek Deveci Kocakoc, Berrin Bercik Inal, Okan Dikker, Cengiz Aydin

**Affiliations:** 1 Department of Medical Biochemistry, University of Health Sciences, Istanbul Training and Research Hospital, 34098 Istanbul, Türkiye; tugaysolar@hotmail.com (T.S.); drberrininal@gmail.com (B.B.I.); 2 Department of Econometrics, Faculty of Economics and Administrative Sciences, Dokuz Eylul University, 35340 Izmir, Türkiye; ipek.deveci@deu.edu.tr; 3 Department of Medical Biochemistry, University of Health Sciences, Istanbul Prof. Dr. Cemil Taşçıoğlu City Hospital, 34384 Istanbul, Türkiye; okan.dikker@sbu.edu.tr; 4 Department of Medical Biochemistry, University of Health Sciences, Kanuni Sultan Suleyman Training and Research Hospital, 34303 Istanbul, Türkiye; drcengiz_aydin@yahoo.com

**Keywords:** albumin-adjusted calcium, corrected calcium, hypoalbuminemia, hypocalcemia, ionized calcium, machine learning, nested cross-validation

## Abstract

**Background/Objectives:** This study aimed to develop and evaluate machine learning models for predicting ionized calcium levels in patients with hypoalbuminemia, compare their performance with classical correction formulas and unadjusted total calcium levels, and assess their transportability through external validation. **Methods:** In a retrospective development cohort of 14,745 adults with hypoalbuminemia, XGBoost and ordinary least squares (OLS) models used total calcium, albumin, total protein, and eGFR and were evaluated with nested cross-validation. Frozen models were then applied without recalibration to an independent external cohort of 2751 patients using the same analyzer platforms. **Results:** Development XGBoost performance was R^2^ = 0.500 and AUC-ROC = 0.798 (95% CI 0.788–0.807), with 98.5% sensitivity and 26.0% specificity. External R^2^ was 0.397 for XGBoost and 0.363 for OLS. External AUC-ROC was 0.818 (95% CI 0.791–0.845) for XGBoost, 0.823 (0.797–0.850) for OLS, and 0.819 (0.791–0.845) for total calcium. Threshold-matched and exploratory decision-curve analyses showed no clear incremental XGBoost advantage over total calcium. **Conclusions:** Machine learning models provided useful continuous ionized-calcium estimates and high sensitivity for hypocalcemia detection, although specificity was low. In independent external validation, hypocalcemia discrimination was comparable to unadjusted total calcium. The models should be considered complementary rather than substitutes for measured ionized calcium, and further multicenter and prospective evaluation is warranted.

## 1. Introduction

Calcium is a mineral that is tightly controlled and important for many bodily functions, including bone mineralization, neuromuscular excitability, and intracellular signal transduction. Problems in calcium homeostasis can have serious health effects and may even be fatal. Therefore, it is important to accurately estimate calcium levels, correctly identify imbalances, and quickly treat low or high calcium levels to ensure appropriate clinical treatment [1]. Calcium in blood is made up of three main components: about 50% is in free (ionized) form, about 40% is bound to plasma proteins, mostly albumin, and about 10% is combined with small anions, such as bicarbonate, phosphate, lactate, and citrate [2]. Ionized calcium is a biologically active calcium component. In contrast, the protein-bound fraction acts as a dynamic reservoir that maintains a stable concentration of ionized calcium over short periods. Albumin is the most abundant protein in circulation and has a high ligand-binding capacity; therefore, changes in albumin concentration can significantly affect the measured total calcium levels without affecting the physiologically active ionized fraction. This situation forms the basis of the phenomenon of pseudohypocalcemia, which can lead to low measured total calcium levels despite normal ionized calcium levels [3,4]. Therefore, albumin-adjusted calcium formulas are commonly used to estimate corrected calcium levels, particularly in hypoalbuminemic conditions [5]. Recent evidence has raised substantial concerns regarding the clinical utility of albumin-adjusted calcium formulas, suggesting that these correction equations do not consistently outperform unadjusted total calcium and may introduce systematic bias [1]. In recent years, data-driven modeling approaches have been proposed as alternative strategies for improving the estimation of ionized calcium.

Recent studies have demonstrated that while conventional methods, such as albumin-adjusted calcium, often fall short in accuracy, particularly in patients with hypoalbuminemia or acid-base disorders, machine learning algorithms show promise in enhancing their predictive capabilities [2,5]. Previous studies have suggested that incorporating additional biochemical variables, along with total calcium and albumin levels, may improve the discrimination of ionized calcium abnormalities. This shift underscores the need for more sophisticated approaches to assess calcium status and supports further investigation of data-driven models as complementary approaches for estimating ionized calcium levels and identifying calcium abnormalities [6]. Although recent studies by Bancal et al. and Kumari et al. applied machine learning approaches to calcium estimation and reported promising results [2,5], both evaluated the model performance within their respective single-center datasets, and the transportability of these approaches to an independent external cohort remained uncertain. Recent guidance has become more critical of routine albumin adjustment: a 2026 joint IOF/IFCC/EFLM position statement recommended against routine reporting of albumin-adjusted (“corrected”) calcium [7]. In parallel, a large retrospective study of 84,410 patients reported that machine learning models based on routinely available biochemical measurements outperformed the conventional correction formulas evaluated in that study for ionized-calcium prediction [8].

This study aimed to develop and evaluate machine learning models for directly predicting measured ionized calcium levels in patients with hypoalbuminemia, compare their performance with that of unadjusted total calcium and conventional calcium correction formulas, and assess their transportability in an independent external cohort. In addition, the diagnostic performance of these approaches in identifying hypocalcemia was evaluated using the measured ionized calcium level as a reference.

## 2. Materials and Methods

### 2.1. Study Population

Patients aged ≥ 18 years with simultaneous measurements of total calcium, albumin, total protein, creatinine, estimated glomerular filtration rate (eGFR), and ionized calcium obtained from samples collected at the same time between February 2024 and October 2025 at Istanbul Training and Research Hospital were included in the study. Data were retrospectively obtained using a laboratory information system (LIS). Individuals under 18 years of age and those whose samples were rejected were excluded from this study. Only the first measurement was included in the analysis of individuals with multiple results. The primary analysis was restricted to patients with hypoalbuminemia, defined as a serum albumin concentration < 35 g/L, with complete data for total calcium, albumin, total protein, eGFR, and ionized calcium (*n* = 14,745) because calcium interpretation is particularly challenging in this population. Because inclusion required an available measured ionized-calcium result obtained on a blood-gas analyzer, the development cohort represents a selected ionized-calcium-tested population rather than all adults with hypoalbuminemia. Ethical approval for this study was granted by the Clinical Research Ethics Committee of Istanbul Training and Research Hospital (approval number: 37, date: 23 January 2026). All phases of this study were designed in accordance with the principles of the Declaration of Helsinki. An internal study and analysis protocol was prepared before the main analyses, but it was not prospectively registered or made publicly available. The study was not prospectively registered.

**Figure 1:**
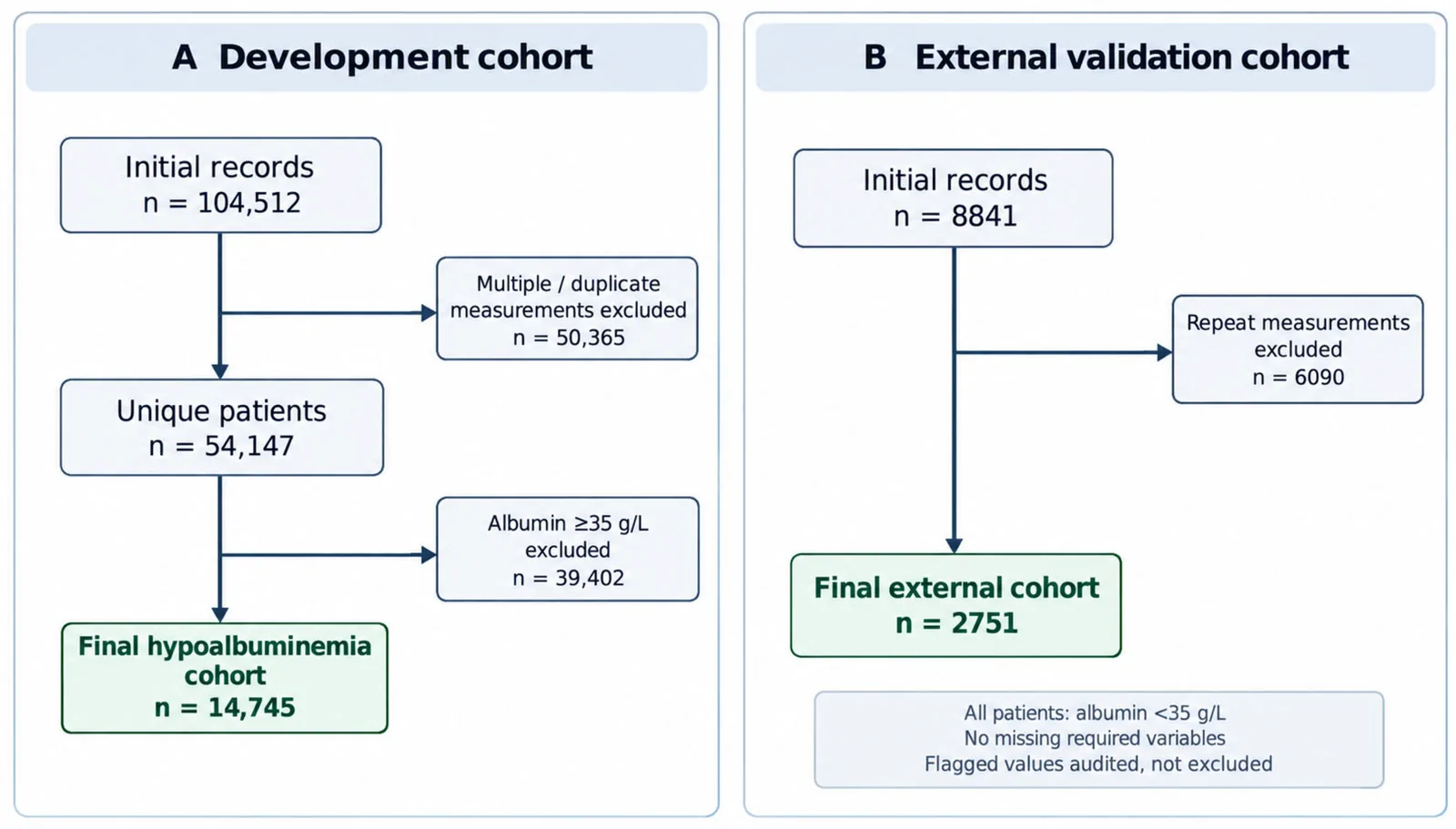
Patient selection in the (**A**) development cohort and (**B**) external validation cohort. Sequential counts are shown for the available source datasets; the external records already met the hypoalbuminemia criterion.

No a priori sample-size calculation was performed because this retrospective study included all eligible patients available in the laboratory information system during the predefined development period. In the development cohort selection dataset, 104,512 records were available. After excluding 50,365 multiple or duplicate measurements, 54,147 unique patients remained. A further 39,402 patients with albumin levels ≥ 35 g/L were excluded, resulting in a final development cohort of 14,745 patients with hypoalbuminemia (Figure 1A).

### 2.2. Laboratory Parameters

Serum total calcium, albumin, total protein, and creatinine levels were assayed using a fully automated chemistry analyzer, AU5800 (Beckman Coulter, Brea, CA, USA). Serum total calcium was measured using the Arsenazo III method, albumin using the bromocresol green method, total protein using the Biuret method, and creatinine using the modified kinetic Jaffe method (Beckman Coulter, Brea, CA, USA). eGFR was calculated using the Chronic Kidney Disease Epidemiology Collaboration creatinine equation [9]. Ionized calcium was measured using a RapidLab 1265 blood gas analyzer (Siemens Healthcare Diagnostics Inc., Tarrytown, NY, USA) using the direct ion-selective electrode method.

### 2.3. Scale Alignment for Formula Comparison

The routinely reported reference interval for total calcium was 2.20–2.65 mmol/L, while the reference interval for ionized calcium measured by blood gas analysis was 1.15–1.35 mmol/L. To compare the methods expressed on different scales, unadjusted total calcium and conventional correction-formula outputs expressed on the total-calcium scale were aligned numerically with the ionized-calcium scale. The exact transformation used in the analyses was ionized-calcium scale = (total-calcium-scale value + 0.386725)/2.2455. The corresponding code-defined total calcium limits were 2.1956 and 2.6447 mmol/L, respectively. This transformation was used only for comparative evaluation and was not intended as a physiological conversion equation. The machine learning models were trained and evaluated directly against the measured ionized calcium, as described in Section 2.5. Supplementary Table S1 distinguishes between two equation types: adjusted total-calcium formulas and equations that directly estimate ionized calcium. Only outputs expressed on the total-calcium scale underwent numerical alignment, and equations already producing ionized calcium were retained on their native output scale.

### 2.4. Published Calcium Equations

In this study, we evaluated several published calcium equations. Some of these equations calculate the adjusted total calcium, whereas others estimate ionized calcium directly. The evaluated equations were Payne [10], Simplified Payne [11], Orrell [12], Berry [13], Thode [14], James [15], Antonio (A), Antonio (B), Antonio (C) [16], Pekar [17], Jain [18], Dent [19], Parfitt [20], Walker [21], Clase [22], and Ridefelt [23]. The equations and their output scales are listed in Supplementary Table S1.

In the main comparisons, we focused on Simplified Payne and Jain. Simplified Payne was included because this formula was routinely used in our LIS for albumin-adjusted calcium. Jain was also included as the main conventional comparator because it has been used in previous calcium studies and showed relatively good performance among the correction formulas evaluated in our development cohort. The other equations were also analyzed and are shown in Supplementary Table S1. This selection was made to keep the main results easier to present. It did not affect model training, hyperparameter tuning, or classification thresholds.

### 2.5. Statistical Analysis and Model Development

All machine learning analyses were conducted in Python (version 3.14.0) using scikit-learn (version 1.7.2) and XGBoost (version 3.1.3) libraries for model development and evaluation. Model interpretability analyses were performed using the SHapley Additive exPlanations (SHAP) library (version 0.50.0). Data preprocessing was performed using the pandas (version 2.3.3) and NumPy (version 2.3.5) libraries.

#### 2.5.1. Data Preprocessing and Outlier Handling

Non-numeric placeholder entries were converted to missing values. For machine learning model development, the predictor variables were total calcium, albumin, total protein, and eGFR, with measured ionized calcium as the target variable. pH and serum creatinine levels were not included as predictors in the final model set. Correlation analysis within the hypoalbuminemia subgroup showed a very strong inverse association between serum creatinine level and eGFR; eGFR was retained because it is a routinely reported renal index that avoids the need for log-transformation of creatinine. pH was also excluded from the final predictor set to restrict the model to routinely available serum biochemical variables and to avoid dependence on an additional blood-gas parameter. Albumin and total protein were moderately correlated (Spearman r ≈ 0.51) but both were retained because each contributed incremental information in subsequent feature-importance analyses.

Extreme outlier values of the continuous predictor variables were identified using Tukey’s interquartile range (IQR) method and were defined as Q1 − 3 × IQR and Q3 + 3 × IQR. Instead of excluding observations beyond these thresholds, the values were winsorized by replacing them with the nearest boundary value, thereby preserving the observations while limiting their disproportionate influence on model training. This approach is particularly advantageous for clinical datasets where extreme measurements may correspond to severe disease states and thus provide important information [24]. For nested cross-validation, winsorization bounds were estimated within each outer training fold and applied to the corresponding training and test folds to limit information leakage. Extremely high ionized calcium values in each outer training fold were capped at the corresponding training-fold 99.7th percentile. The measured ionized calcium values in the held-out test folds were not capped, winsorized, or otherwise modified, and all out-of-fold performance metrics were calculated against the original uncapped test outcomes. The lower ionized calcium values were left unchanged. For the final full-cohort model used for interpretability analyses and subsequent external validation, winsorization bounds were estimated from the complete hypoalbuminemia cohort. Within nested cross-validation, all preprocessing parameters were estimated exclusively from the corresponding training data and were subsequently applied unchanged to the held-out test fold.

#### 2.5.2. Feature Definition

The primary comparison of the machine learning algorithms used the same predictor set: total calcium, albumin, total protein, and eGFR. These variables are routinely available in clinical laboratories, and with the exception of the ionized calcium reference measurement used for model development and evaluation, they do not require dedicated blood gas sampling. This predictor set was selected based on clinical–physiological relevance and the correlation analysis described in Section 2.5.1, which guided the exclusion of pH and serum creatinine to reduce redundancy and limit multicollinearity. For the present study, the intended-use population was adults with hypoalbuminemia who had routine serum biochemistry available. The models were evaluated as possible laboratory decision-support tools for selective confirmatory ionized-calcium testing, not for population-wide screening or as replacements for measured ionized calcium.

#### 2.5.3. Nested Cross-Validation, Hyperparameter Tuning, and Regression Metrics

Model comparison was performed using nested cross-validation with 10 outer folds and five inner folds (random seed 42). Within each outer training fold, hyperparameters were selected by grid search, maximizing the negative root mean squared error (RMSE) on the inner folds, and the selected configuration was then evaluated on the corresponding held-out outer test fold. Ordinary least-squares (OLS) regression had no tunable hyperparameters and served as a transparent baseline. Ridge, LASSO, and Elastic Net regression were implemented with classical z-score standardization, z = (x − μ)/σ, with μ and σ estimated exclusively from the training fold within a pipeline framework to prevent data leakage. Random Forest and XGBoost hyperparameter grids included tree depth, number of estimators, learning rate (XGBoost), subsampling, and related regularization parameters, predefined to balance model complexity and generalization. For OLS, no inner-fold grid search was performed, and a separate model was fitted on each outer training fold and evaluated on its held-out outer test fold.

Model performance was assessed using multiple complementary metrics. The coefficient of determination (R^2^) was used to quantify the proportion of variance explained by the model. The mean absolute error (MAE) represents the average size of the prediction errors, whereas the RMSE is more sensitive to large errors. The systematic prediction error was evaluated using bias, defined as the mean difference between the predicted and observed values, and percentage bias (bias%), which expresses this difference relative to the observed values. The performance was summarized as (i) the mean ± SD across outer folds and (ii) metrics computed on the concatenated out-of-fold predictions, together with 95% bootstrap confidence intervals (2000 resamples, percentile method, fixed random seed of 42). For classification, ROC, and Bland–Altman analyses involving XGBoost, continuous predictions were concatenated outer-fold out-of-fold predictions from the same nested cross-validation (each test observation predicted by a model trained only on its outer training fold, with hyperparameters selected by the corresponding inner-fold grid search). A final XGBoost model was fitted to the complete development cohort using the hyperparameter set most frequently selected across the outer folds (max_depth = 4, learning_rate = 0.05, *n*_estimators = 100, subsample = 0.8, colsample_bytree = 1.0). This model was used for post-hoc interpretability analyses and was subsequently applied without modification in the external validation cohort. The selection frequency was counted for each complete hyperparameter combination, not as separate modes of individual parameters. The selected complete combination occurred in three of the 10 outer folds; the frequency rule provided a single final configuration and did not establish statistical optimality (Supplementary Table S2).

#### 2.5.4. Formula Evaluation Scale

To enable direct comparison with the machine learning models, unadjusted total calcium and classical correction-formula outputs expressed on the total-calcium scale were converted to the ionized-calcium scale using the transformation described in Section 2.3 and compared with the measured ionized calcium. Accordingly, the MAE, RMSE, and bias were expressed on the ionized-calcium scale. Error measures for conventional formulas after alignment are conditional on this transformation and do not indicate a direct physiological prediction of ionized calcium by these formulas. The measured ionized calcium level remained the common reference outcome for all methods.

#### 2.5.5. Classification Performance

Reference calcium categories were defined from measured ionized calcium using the laboratory reference limits: hypocalcemia (<1.15 mmol/L), normocalcemia (≥1.15 to <1.35 mmol/L), and hypercalcemia (≥1.35 mmol/L). The continuous XGBoost out-of-fold predictions, which were on the ionized-calcium scale, were categorized using the same thresholds. Because the unadjusted total calcium and classical correction formulas are expressed on the total-calcium scale, they were categorized using the exact aligned total-calcium limits of 2.1956 and 2.6447 mmol/L (Section 2.3). The original code classified values below 2.1956 as hypocalcemic, values above 2.6447 as hypercalcemic, and values at either boundary as normocalcemic. The same exact thresholds were used in development and external validation; the routinely reported total-calcium interval was 2.20–2.65 mmol/L. The lower limit of 1.15 mmol/L was used because it is the laboratory reference limit for ionized calcium; it was not selected to maximize model performance.

The classification performance for the detection of hypocalcemia was evaluated using a one-vs-rest scheme, with hypocalcemia defined as a positive class. For each method (unadjusted total calcium, Jain and Simplified Payne adjusted calcium formulas, and XGBoost model), clinical sensitivity, clinical specificity, positive and negative predictive values (PPV, NPV), and F1-score were calculated. Threshold-independent discrimination was quantified using the area under the receiver operating characteristic curve (AUC-ROC) computed from the continuous calcium values (predicted values for XGBoost and measured/calculated values for total calcium and the correction formulas) as the ranking score. Because lower calcium values indicate a higher probability of hypocalcemia, the negative value of each continuous calcium estimate was used as the ROC ranking score. Ninety-five percent confidence intervals for proportion-based metrics were calculated using the Wilson score method, whereas confidence intervals for the AUC were estimated using nonparametric bootstrap resampling (2000 resamples, percentile method, fixed random seed of 42). Because more than 80% of the hypoalbuminemic patients were hypocalcemic according to the ionized calcium, the overall accuracy was affected by class imbalance and was therefore reported only as a complementary measure; discrimination was interpreted primarily on the basis of the AUC together with clinical sensitivity and specificity. Because the machine learning models were trained as regression models to predict continuous ionized calcium values, no class-balancing procedure was applied during model development. Class imbalance was considered when interpreting the secondary classification analysis by jointly evaluating the AUC-ROC, clinical sensitivity, clinical specificity, PPV, NPV, and F1-score. Balanced accuracy, defined as the mean of sensitivity and specificity, and the Matthews correlation coefficient (MCC) calculated from all four binary confusion-matrix cells were also reported as complementary measures in this imbalanced cohort (Supplementary Table S3).

#### 2.5.6. Model Interpretability

To enhance clinical interpretability, feature-level contributions to the final selected model were examined using SHAP. Rooted in cooperative game theory, SHAP quantifies each feature’s marginal contribution to a prediction by averaging its effect across all possible feature subsets, ensuring desirable properties, such as local accuracy and consistency, that distinguish it from alternative attribution methods [25,26]. A key property of this framework is that the sum of all assigned SHAP values equals the difference between the model’s actual prediction and the baseline output, whereas features with no predictive impact receive an attribution of zero [27]. SHAP values were computed for the final XGBoost model trained on the hypoalbuminemia cohort using total calcium, albumin, total protein, and eGFR as predictors and measured ionized calcium as the outcome. Model explainability was evaluated using SHAP summary plots, which quantify both the relative importance of each predictor and the direction of its effect on model output. Each point in the plot represents an individual observation. The horizontal position (SHAP value) indicates the extent to which a given feature increases or decreases the predicted ionized calcium value, whereas the color encodes the magnitude of the feature itself, with red indicating higher values and blue indicating lower values.

#### 2.5.7. Agreement Analysis

Bland–Altman plots were used to compare each method with the measured ionized calcium. XGBoost used nested cross-validation out-of-fold predictions on the native ionized-calcium scale. For the Bland–Altman analysis, total calcium and the correction-formula outputs were transformed to the ionized-calcium scale using the same transformation applied in the scale-aligned regression comparison described in Section 2.5.4.

#### 2.5.8. Ordinary Least Squares Equation

As a transparent linear alternative, the final OLS equation predicting measured ionized calcium from total calcium, albumin, total protein, and eGFR was fitted to the complete hypoalbuminemia development cohort (*n* = 14,745). Predictor values were winsorized using development-derived 3 × IQR bounds, and the training target was capped at the upper 99.7th percentile. The resulting coefficients were rounded off for presentation and external applications. The internal performance of the OLS modeling procedure was estimated separately from fold-specific out-of-fold predictions during nested cross-validation, rather than from the exact final coefficient vector.

#### 2.5.9. Methodological Consistency

All preprocessing, modeling, and evaluation steps were applied consistently across the analyses. Identical predictor definitions, winsorization rules, evaluation metrics, and random seeds were used throughout the nested cross-validation. Feature importance and SHAP were obtained from the final full-cohort XGBoost refit described in Section 2.5.3, and the same frozen model was applied without modification to the external validation cohort. Classification, ROC, and Bland–Altman analyses for XGBoost in the development cohort used out-of-fold predictions to avoid optimistic reuse of training observations, ensuring the comparability of results within the hypoalbuminemia cohort.

#### 2.5.10. pH Sensitivity Analysis

For the sensitivity analysis, we compared the original four-variable XGBoost model (total calcium, albumin, total protein, and eGFR) with an extended model that also included blood-gas pH. Both models were developed on the same pH-complete subset of the development cohort (*n* = 14,741) using identical 10-outer-fold and five-inner-fold cross-validation splits (random seed 42), the original hyperparameter grid, and the same preprocessing and evaluation procedures as the primary analysis. Missing pH values were not imputed. Out-of-fold R^2^, MAE, RMSE, and bias values were compared and paired differences were evaluated using 2000 patient-level bootstrap resamples (95% percentile confidence intervals). Hypocalcemia classification used the fixed ionized-calcium threshold of <1.15 mmol/L. The original four-variable model remained the primary model and was the only one evaluated in the external cohort.

In a further exploratory analysis, the saved out-of-fold predictions from this pH-complete development subset were assessed for acidemia (pH < 7.35), normal pH (7.35–7.45, inclusive), and alkalemia (pH > 7.45). Both the model specifications and fixed hypocalcemia threshold were unchanged, and no subgroup-specific retraining was performed (Supplementary Table S4).

#### 2.5.11. Predictor Ablation Analysis

An ablation analysis compared total calcium alone with the original four-variable XGBoost model and two intermediate predictor sets in the same 14,745 development patients. All models used identical nested cross-validation splits and the original modeling protocol. Paired differences were evaluated using 2000 bootstrap replicates. The full methods and results are provided in the Supplementary Methods and Tables S5–S7.

### 2.6. External Validation

The external validation cohort consisted of adult patients with hypoalbuminemia (albumin < 35 g/L) evaluated at an independent center between February 2024 and December 2025. The external center used the same analytical platforms and measurement methodologies as the development center, with biochemical measurements performed using a Beckman Coulter AU5800 analyzer (Beckman Coulter, Brea, CA, USA) and ionized calcium measured using a Siemens RAPIDLab 1265 blood gas analyzer (Siemens Healthcare Diagnostics Inc., Tarrytown, NY, USA). Records were ordered according to patient identifier, collection date, and sample number, and only the first chronologically eligible measurement per patient was retained. The eligibility criteria, predictor definitions, and calcium reference intervals were consistent with those applied in the development cohort. Total calcium, albumin, total protein, creatinine, and ionized calcium levels were measured using the same analyzer models and analytical methodologies as in the development cohort.

The external selection dataset contained 8841 measurement records. After retaining only the chronological first measurement for each patient, 6090 subsequent or duplicate records were removed, leaving 2751 unique patients (Figure 1B). All records in the supplied external dataset met the albumin level < 35 g/L criterion. There were no missing values for the required predictors or measured ionized calcium; therefore, no further records were excluded for missingness. Suspicious-value flags were reviewed but were not used as exclusion criteria. The external validation cohort similarly included all eligible patients available during the predefined validation period.

The frozen XGBoost regression model and the full-cohort OLS equation, both developed for the hypoalbuminemia cohort described above, were subsequently evaluated in this independent external hypoalbuminemia cohort. Both models were applied without refitting, hyperparameter tuning, recalibration, or threshold optimization. For external applications, the XGBoost model was the single final fit for the complete development cohort (*n* = 14,745) using the hyperparameter set most frequently selected across the outer nested cross-validation folds: objective = reg:squarederror, max_depth = 4, learning_rate = 0.05, *n*_estimators = 100, subsample = 0.8, colsample_bytree = 1.0, and random_state = 42. During this full-cohort fit, the training target was capped at the development cohort 99.7th percentile of measured ionized calcium (quantile 0.997); values below this upper bound were left unchanged. The measured ionized calcium values in the external cohort were not capped, winsorized, or otherwise modified. Predictor winsorization limits were derived exclusively from the development cohort using Tukey’s 3 × IQR bounds (Section 2.5.1) and were applied unchanged to the external predictors (total calcium, albumin, total protein, and eGFR). The OLS model was applied using a previously reported full-development equation without re-estimation of the coefficients: ionized calcium = 0.339 + 0.438 × total calcium − 0.00441 × albumin − 0.00114 × total protein + 0.000051 × eGFR. The unadjusted total calcium level was included only as a routine clinical benchmark for calcium classification. Classical adjusted calcium formulas were not re-evaluated in the external cohort because the objective of this analysis was to assess the transportability of the newly developed prediction models rather than to re-validate the classical correction equations already characterized in the development cohort.

External regression performance was summarized using R^2^, MAE, RMSE, and bias, where bias was defined as the mean (predicted − observed) in mmol/L. External calibration was assessed by the linear model observed ionized calcium = intercept + slope × predicted ionized calcium and by Bland–Altman analysis of predicted minus measured ionized calcium. For hypocalcemia detection, the reference positive class was defined as measured ionized calcium < 1.15 mmol/L. Predicted hypocalcemia was defined as predicted ionized calcium < 1.15 mmol/L for XGBoost and OLS, and as unadjusted total calcium < 2.1956 mmol/L for the total-calcium benchmark (the exact code-defined cutoff). Three-class ionized-calcium categories used the development-cohort reference thresholds (hypocalcemia < 1.15 mmol/L; normocalcemia ≥ 1.15 to <1.35 mmol/L; hypercalcemia ≥ 1.35 mmol/L); for the total-calcium benchmark the exact code-defined limits were 2.1956 and 2.6447 mmol/L (Section 2.3). The diagnostic metrics included clinical sensitivity, clinical specificity, predictive values, F1-score, accuracy, and AUC-ROC. Because a lower predicted calcium indicates a higher likelihood of hypocalcemia, the AUC-ROC used the negative of the continuous calcium value as the ranking score (−predicted ionized calcium for XGBoost and OLS; −total calcium for the benchmark). Ninety-five percent confidence intervals for the regression metrics, AUC-ROC, and related continuous summaries were estimated using nonparametric bootstrap resampling (2000 resamples, percentile method, random seed 42). Ninety-five percent confidence intervals for the sensitivity, specificity, PPV, and NPV were calculated using the Wilson score method. For the paired external comparison of XGBoost and OLS, differences in R^2^, MAE, and RMSE were estimated using a patient-level nonparametric bootstrap (2000 resamples, seed 42). Both models were evaluated using identical patient indices for each resample, and 95% confidence intervals were calculated using the percentile method.

For a formal comparison of overall hypocalcemia discrimination in the external cohort, correlated ROC-AUCs for XGBoost, OLS, and unadjusted total calcium were compared using paired DeLong tests. The three two-sided *p*-values were adjusted using the Holm procedure (α = 0.05). The DeLong 95% confidence intervals for individual AUCs and paired differences are shown in Supplementary Table S8.

To examine whether the high sensitivity of XGBoost at the fixed 1.15 mmol/L cutoff was mainly related to the selected operating point, we performed a threshold-matched analysis against unadjusted total calcium. Two alternative XGBoost thresholds were selected using only development-cohort out-of-fold predictions: <1.0872 mmol/L to match the development specificity of total calcium (60.8%) and <1.0853 mmol/L to match its development sensitivity (80.8%). These thresholds were then frozen and applied unchanged to the external cohort. No external outcome was used for threshold selection. Paired external differences in sensitivity and specificity were estimated with 2000 patient-level bootstrap resamples (seed 42; 95% percentile confidence intervals). Full results are provided in Supplementary Table S12.

For decision-curve analysis, the continuous calcium scores were converted to hypocalcemia probabilities using separate univariable logistic mappings fitted only in the development cohort. The score was the negative continuous calcium value (XGBoost or OLS out-of-fold predicted ionized calcium, or observed unadjusted total calcium), so lower calcium indicated higher hypocalcemia probability. These development-derived mappings were frozen and applied unchanged to the external cohort, without external recalibration or threshold optimization. Net benefit was evaluated for a hypothetical strategy of triggering confirmatory ionized-calcium measurement at threshold probabilities from 0.05 to 0.95, comparing XGBoost, OLS, unadjusted total calcium, treat-all, and treat-none. Paired patient-level bootstrap resampling (2000 resamples; seed 42) was used for 95% confidence intervals of net-benefit differences at selected thresholds. Because no harm ratio for missed hypocalcemia versus unnecessary confirmatory testing was prespecified, this analysis was exploratory (Supplementary Table S13 and Figure S4).

As an exploratory external-validation subgroup analysis, the original frozen predictions were evaluated within albumin categories (<25, 25–<30, and 30–<35 g/L) and eGFR categories (<30, 30–<60, and ≥60 mL/min/1.73 m^2^), using observed values to define each group. The XGBoost and OLS regression metrics and fixed-threshold classification/ROC metrics for XGBoost, OLS, and unadjusted total calcium are summarized without model refitting or threshold optimization (Supplementary Table S9). This subgroup analysis used the original fixed thresholds and did not involve external threshold optimization.

Model explainability in the independent external cohort was also assessed using the SHAP TreeExplainer applied to the saved final XGBoost model used for external prediction, without retraining or retuning. The SHAP values were calculated for all 2751 external observations after applying the development-derived 3 × IQR predictor winsorization bounds stored with the model. Global feature importance was summarized as the mean absolute SHAP value across patients. Relative shares were obtained by normalizing these means to sum to 100% and were descriptive only.

## 3. Results

### 3.1. Development Cohort

The primary analysis included 14,745 observations with complete data for total calcium, albumin, total protein, eGFR, and the reference outcome variable (measured ionized calcium). The mean age of the patients was 68.7 ± 15.7 years, and 53.8% were male. The descriptive statistics for all continuous variables are summarized in Table 1. Mean measured ionized calcium was 1.06 ± 0.13 mmol/L. The mean total calcium concentration was 2.09 ± 0.23 mmol/L. The mean albumin concentration was 29.0 ± 4.27 g/L.

Table 2 summarizes the nested cross-validated regression performance of the machine learning models for predicting the measured ionized calcium levels in the hypoalbuminemia cohort. XGBoost showed the highest out-of-fold performance (R^2^ = 0.500, 95% CI 0.477–0.522; MAE = 0.066; RMSE = 0.092), followed closely by Random Forest (R^2^ = 0.496, 95% CI 0.474–0.517). OLS, Ridge, LASSO, and Elastic Net yielded nearly identical performance (R^2^ ≈ 0.458; MAE ≈ 0.068; RMSE ≈ 0.096), with bias close to zero for all models (Table 2).

In the pH-complete subset (*n* = 14,741), the four-variable XGBoost model exhibited an out-of-fold R^2^ value of 0.501, MAE of 0.0658 mmol/L, and RMSE of 0.0924 mmol/L. With pH included, the corresponding values were 0.511, 0.0644, and 0.0915 mmol/L, respectively. The paired differences (pH-extended minus four-variable model) were 0.0094 (95% CI 0.0063–0.0130) for R^2^, −0.00140 mmol/L (95% CI −0.00163 to −0.00117) for MAE, and −0.00088 mmol/L (95% CI −0.00120 to −0.00059) for RMSE. At the fixed hypocalcemia threshold, sensitivity was 98.5% for both models (179 vs. 187 false negatives without and with pH), while specificity increased from 25.8% to 27.3% (1909 vs. 1871 false positives). The ROC-AUC was 0.799 without pH and 0.802 with pH. These findings were obtained from internal out-of-fold predictions and were not externally validated using a pH-extended model.

In the exploratory pH strata (*n* = 3130, 7727, and 3884), the four-variable model had a higher MAE in acidemia (0.0685 mmol/L) and alkalemia (0.0738 mmol/L) than in normal pH (0.0608 mmol/L). It underestimated the measured ionized calcium in acidemia (mean bias −0.0187 mmol/L) and overestimated it in alkalemia (+0.0128 mmol/L). Adding pH reduced MAE in all three groups (ΔMAE, pH-extended minus original: −0.0025, −0.0005, and −0.0024 mmol/L, respectively) and reduced the mean bias in both abnormal pH groups (−0.0014 and +0.0007 mmol/L). At the fixed threshold, false negatives increased from 27 to 58 in acidemia but decreased from 55 to 35 in alkalemia (Supplementary Table S4). These were exploratory within-development-cohort findings, and no formal between-group interaction test was performed.

In the development-cohort ablation analysis, the four-variable XGBoost model showed a small improvement in out-of-fold continuous prediction compared to the total-calcium-only XGBoost model (R^2^, 0.500 vs. 0.483; MAE, 0.0659 vs. 0.0676 mmol/L). The paired difference in R^2^ was 0.0169 (95% CI, 0.0128–0.0210). The ROC-AUC did not improve (0.798 vs. 0.803). The results for all four predictor sets, paired comparisons, and fixed-threshold classification are provided in Supplementary Tables S5–S7.

Jain (R^2^ = 0.361; MAE = 0.079; bias = 0.046 mmol/L) and unadjusted total calcium (R^2^ = 0.351; MAE = 0.079; bias = 0.042 mmol/L) showed comparatively better performance among the conventional approaches, whereas the Simplified Payne formula showed a larger positive bias (0.140 mmol/L). Because the machine learning models in Table 2 and classical formulas in Table 3 are presented on the same ionized-calcium scale, their performance metrics can be compared directly. OLS (R^2^ = 0.458; MAE = 0.068; bias = −0.00048 mmol/L) and XGBoost (R^2^ = 0.500; MAE = 0.066; bias = −0.00052 mmol/L) showed lower absolute errors and biases closer to zero than the classical formulas (Table 2 and Table 3).

**Figure 2:**
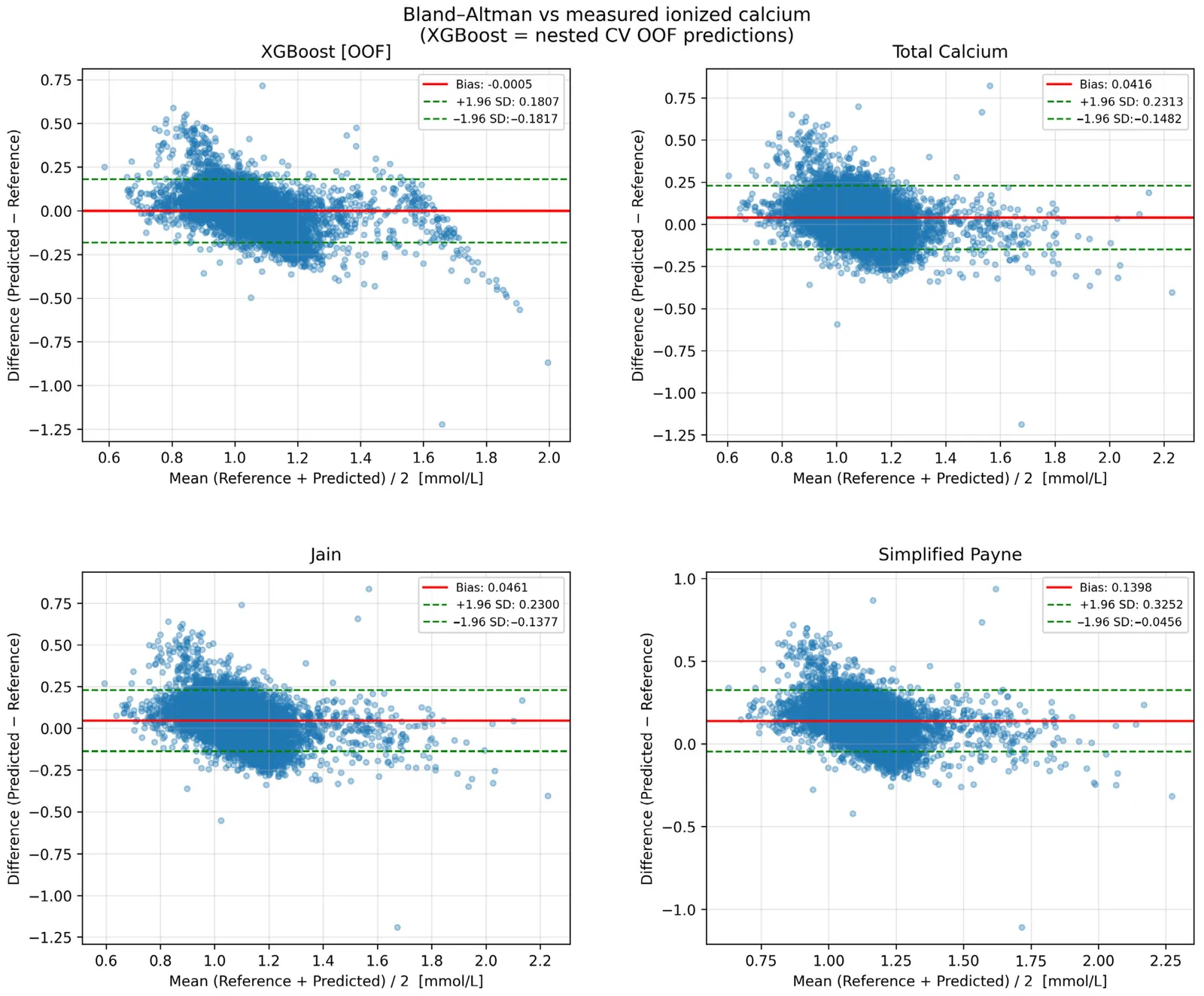
Bland–Altman plots comparing calcium estimation methods with ionized calcium in patients with hypoalbuminemia. The solid red line represents the mean bias, whereas the dashed green lines indicate the 95% limits of agreement (mean bias ± 1.96 SD).

In the Bland–Altman analysis against ionized calcium (Figure 2), the XGBoost out-of-fold predictions demonstrated near-zero bias (−0.0005 mmol/L) with 95% limits of agreement (LoA) of −0.182 to 0.181 mmol/L. After transformation to the ionized-calcium scale, the unadjusted total calcium showed a bias of 0.042 mmol/L (LoA −0.148 to 0.231), the Jain formula showed a bias of 0.046 mmol/L (LoA −0.138 to 0.230), and the Simplified Payne formula showed the highest bias, 0.140 mmol/L (LoA −0.046 to 0.325). Therefore, the XGBoost model showed the smallest bias and narrowest limits of agreement among the evaluated methods.

**Figure 3:**
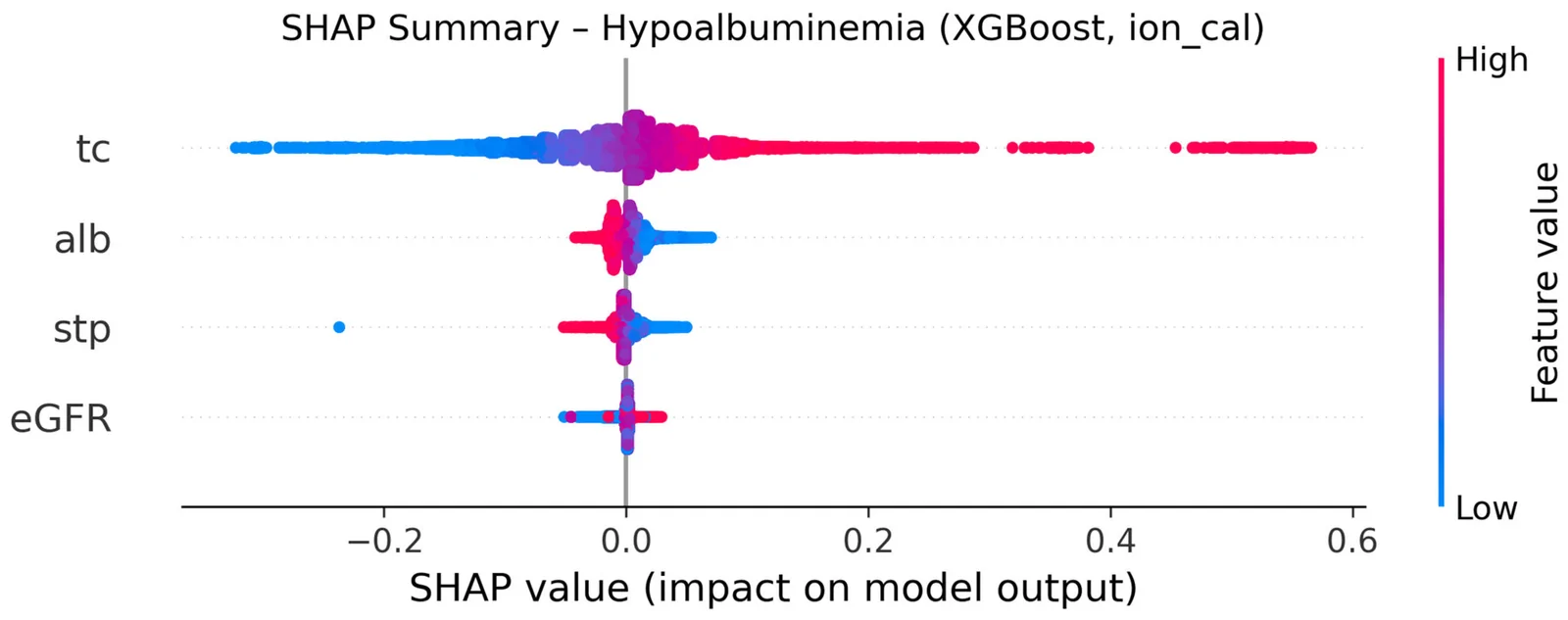
SHAP summary plot for patients with hypoalbuminemia.

Built-in XGBoost gain-based importance ranked total calcium as the dominant predictor (gain 85.5%), followed by albumin (6.3%), total protein (4.7%), and eGFR (3.5%). The SHAP analysis showed the same ranking, with total calcium exhibiting the widest range of SHAP values among the evaluated predictors, followed by albumin, total protein, and eGFR. Higher total calcium values were associated with higher predicted ionized calcium values, whereas albumin, total protein, and eGFR provided smaller corrective contributions to the prediction (Figure 3).

Reference calcium categories were defined based on measured ionized calcium using the laboratory reference interval for hypocalcemia (<1.15 mmol/L), normocalcemia (≥1.15 to <1.35 mmol/L), and hypercalcemia (≥1.35 mmol/L) (Table 4). According to the reference classification, 12,171 (82.5%) patients were categorized as hypocalcemic, 2245 (15.2%) as normocalcemic, and 329 (2.2%) as hypercalcemic. Using total calcium alone, the overall agreement with the reference calcium categories was 76.2%. Misclassification was particularly prominent in the normocalcemia category, where only 53.7% of the cases were correctly classified and 44.2% were incorrectly assigned to hypocalcemia. The application of the Jain formula resulted in a modest increase in the overall agreement (77.6%); however, the misclassification pattern remained largely unchanged, with persistent overclassification of hypocalcemia. Among normocalcemic individuals, only 51.9% were correctly classified using the Jain formula, whereas 45.8% were misclassified as hypocalcemic, closely mirroring the pattern observed for total calcium. Using nested cross-validation out-of-fold predictions, the XGBoost model achieved a higher overall accuracy of 85.1% compared with 76.2% for total calcium and 77.6% for the Jain formula (Table 4).

**Figure 4:**
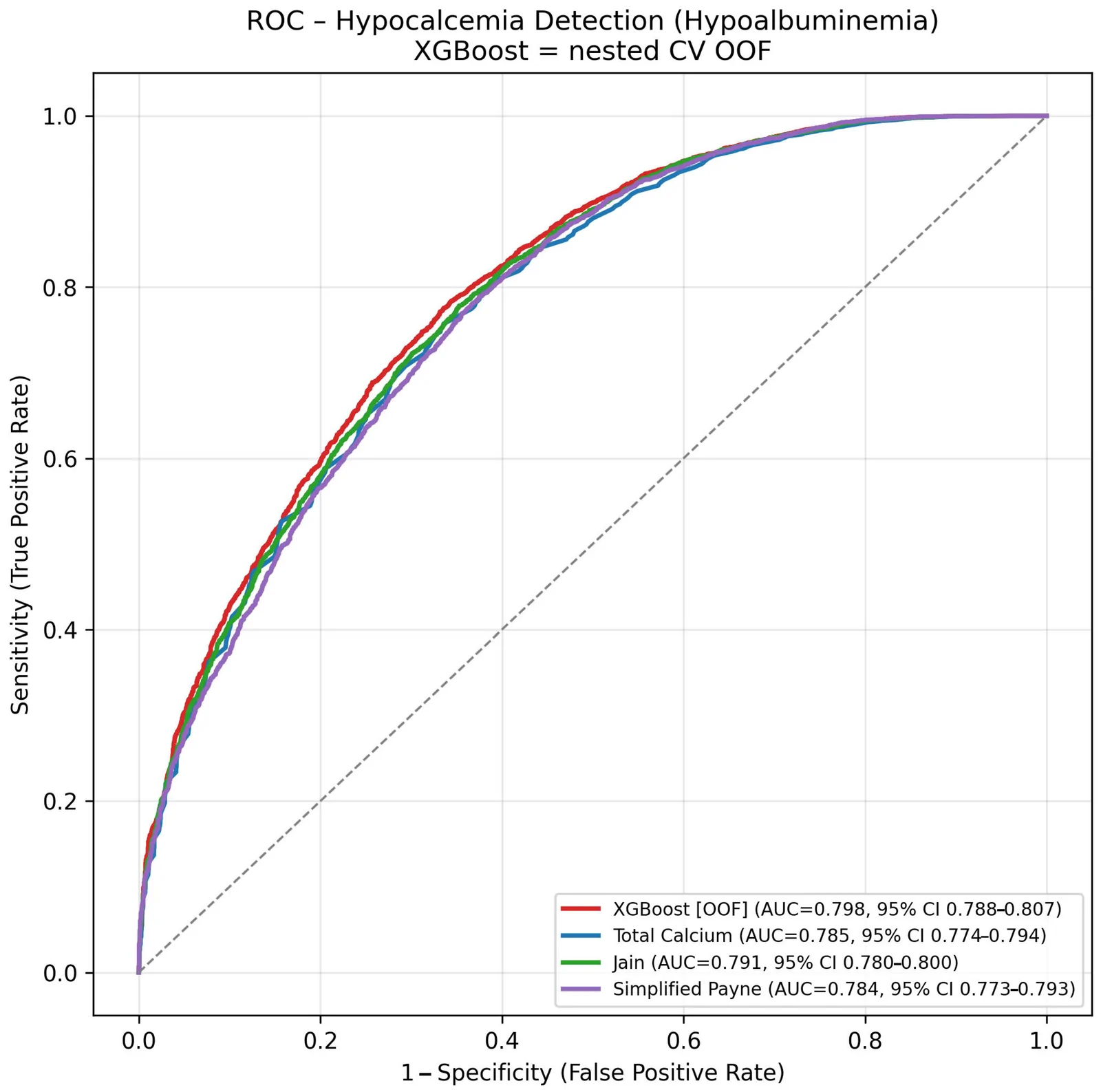
Receiver operating characteristic curves for hypocalcemia detection in patients with hypoalbuminemia.

For hypocalcemia detection, out-of-fold XGBoost showed the highest AUC-ROC (0.798, 95% CI 0.788–0.807) and F1-score (92.0), followed by the Jain formula (AUC-ROC 0.791, 95% CI 0.780–0.800; F1-score 86.5), total calcium (AUC-ROC 0.785, 95% CI 0.774–0.794; F1-score 85.5), and the Simplified Payne formula (AUC-ROC 0.784, 95% CI 0.773–0.793; F1-score 44.2) (Table 5 and Figure 4). The clinical sensitivities for hypocalcemia were 98.5% for XGBoost, 80.8% for total calcium, and 82.7% for the Jain formula. The corresponding numbers of false-negative classifications were 182, 2337, and 2105, respectively. The number of false positives was 1906 for XGBoost, 1010 for total calcium, and 1048 for the Jain formula. The Simplified Payne formula showed the lowest overall accuracy (40.2%) among the evaluated methods. Although it demonstrated high clinical specificity (94.6%) and positive predictive value (96.2%), its clinical sensitivity for detecting hypocalcemia was markedly low (28.7%), indicating that a substantial proportion of hypocalcemic cases were misclassified as normocalcemic. XGBoost had the highest AUC-ROC, F1-score, and clinical sensitivity, together with the lowest clinical specificity (26.0%, 95% CI 24.3–27.7%), among the evaluated methods (Table 5). The corresponding binary confusion matrices for hypocalcemia detection are shown in Supplementary Figure S1.

The OLS equation predicting ionized calcium from total calcium, albumin, total protein, and eGFR was additionally fit on the hypoalbuminemia cohort: ionized calcium = 0.339 + 0.438 × total calcium − 0.00441 × albumin − 0.00114 × total protein + 0.000051 × eGFR. Internal performance was estimated separately using nested cross-validation, yielding an out-of-fold R^2^ of 0.458 (95% CI, 0.437–0.476), MAE = 0.068, and RMSE = 0.096, with negligible bias. The OLS model showed a lower absolute error and bias closer to zero than the classical fixed-coefficient formulas (Table 2 and Table 3). These internal metrics reflect separately fitted outer-fold OLS models rather than a held-out evaluation of the exact final coefficients. The published rounded equation was applied to the independent external cohort after development-derived predictor winsorization without refitting.

### 3.2. External Validation Results

To assess the transportability of the development-cohort models, the frozen XGBoost model and full-cohort OLS equation were applied without modification to an independent external cohort (*n* = 2751) from a separate hospital. Table 6 summarizes the external cohort characteristics, and Table 7 compares the external cohort with the development cohort using standardized mean differences (SMD).

The external cohort had a somewhat lower total protein level and a higher eGFR than the development cohort, and hypocalcemia was more frequent (89.7% vs. 82.5% by the ionized-calcium reference). These distributional differences are formally quantified in Table 7. Total protein and eGFR showed the largest between-cohort differences (|SMD| > 0.30). Hypocalcemia prevalence also differed between cohorts (|SMD| = 0.209). Differences in the remaining variables were smaller, with total calcium, albumin, and ionized calcium levels showing relatively similar distributions between the cohorts (|SMD| ≤ 0.13). Age and sex distributions were comparable between the development and external cohorts (mean age 68.7 ± 15.7 versus 67.7 ± 16.7 years; 53.8% versus 52.6% male), although age and sex were not used as model predictors in either cohort. Development-derived winsorization bounds were applied to the external predictors without further modification; the full winsorization diagnostics are provided in Supplementary Table S10.

**Figure 5:**
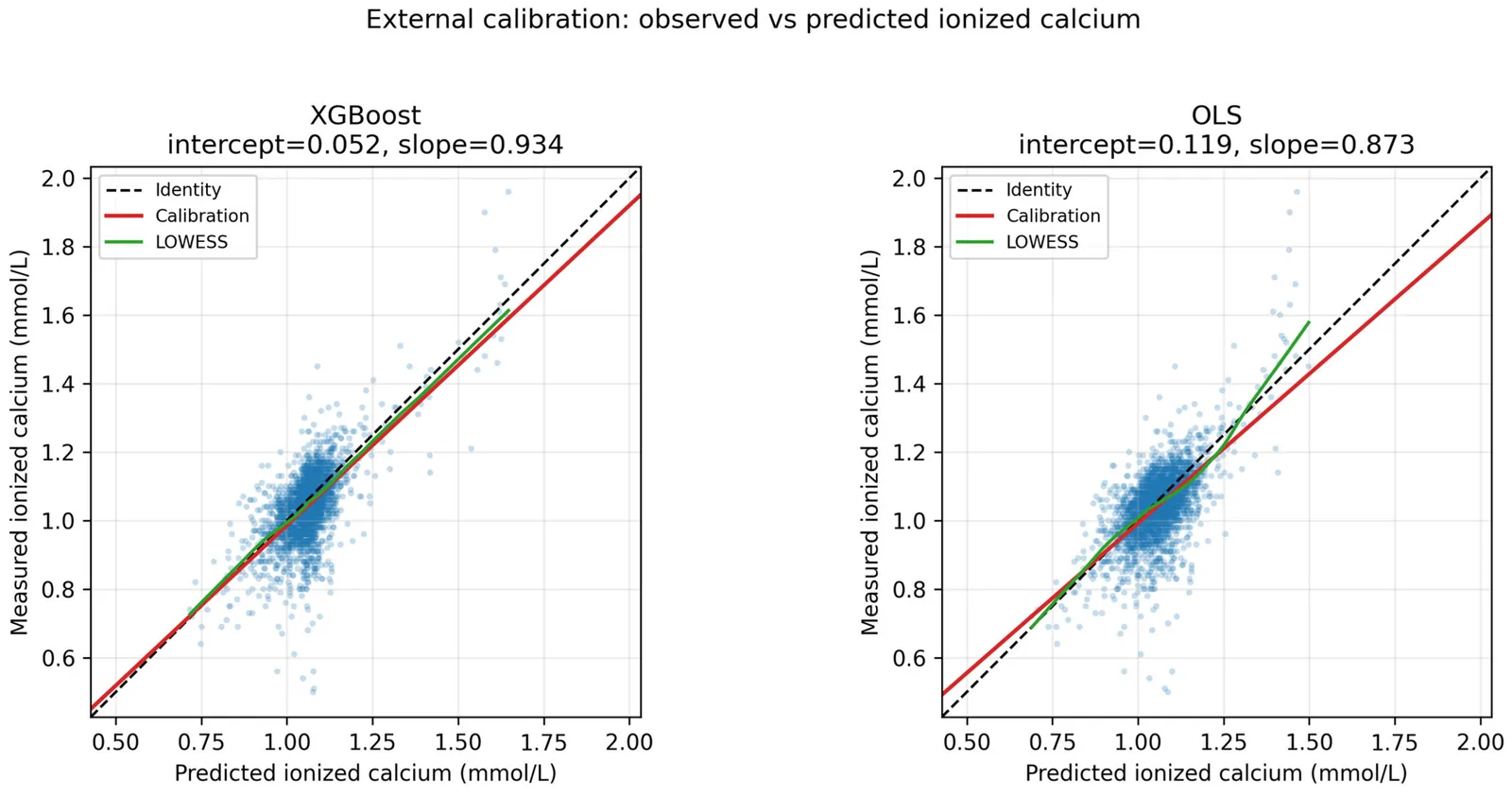
Calibration plots of the XGBoost and OLS models in the external validation cohort. Solid black line: identity; red line: linear calibration; green line: LOWESS smooth.

**Figure 6:**
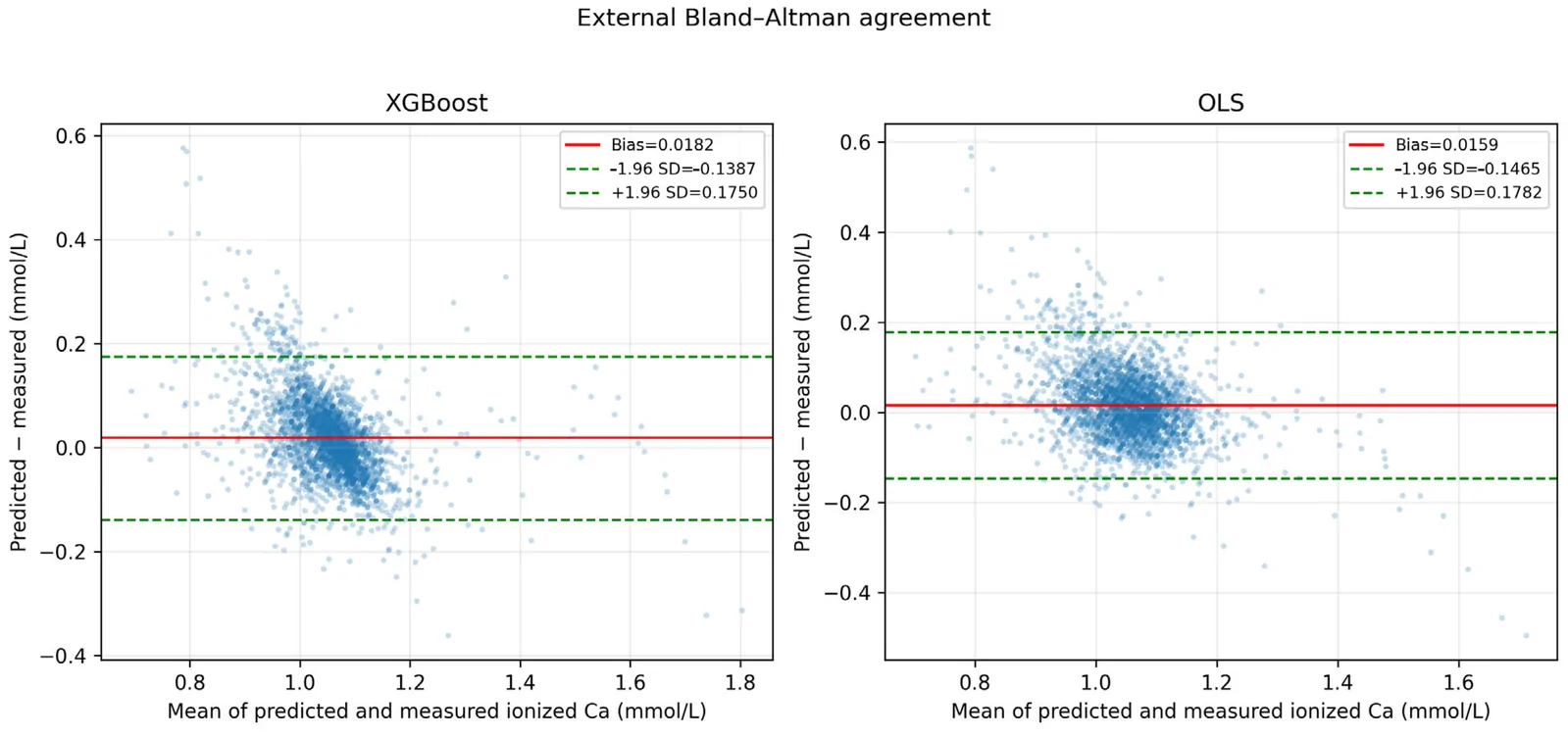
Bland–Altman plots of XGBoost and OLS models in the external validation cohort.

Regression performance was lower in the external cohort than in the development out-of-fold testing for both models: R^2^ fell from 0.500 to 0.397 for XGBoost and from 0.458 to 0.363 for OLS. Both models showed a small systematic positive bias of approximately 0.02 mmol/L, in contrast to the near-zero bias observed in development, and calibration slopes below 1 (0.934 for XGBoost, 0.873 for OLS), indicating that the frozen predictions were modestly more dispersed than the observed ionized calcium values (Table 8 and Figure 5). Despite the lower R^2^, absolute prediction errors were smaller in the external cohort than in the development cohort for both models (MAE 0.059 and 0.060 mmol/L versus 0.066 and 0.068 mmol/L, respectively), reflecting the narrower spread of measured ionized calcium in this cohort (Table 2 and Table 8). The Bland–Altman analysis in the external cohort showed a small positive mean difference for both models, with limits of agreement of −0.139 to 0.175 mmol/L for XGBoost and −0.146 to 0.178 mmol/L for OLS (Figure 6).

In the paired external comparison, the XGBoost-minus-OLS difference was 0.0337 (95% CI, 0.0172–0.0512) for R^2^, −0.00175 mmol/L (95% CI, −0.00251 to −0.00100) for MAE, and −0.00226 mmol/L (95% CI, −0.00362 to −0.00113) for RMSE. These differences indicated a slightly better continuous prediction with XGBoost, although the absolute reductions in the prediction error were small.

**Figure 7:**
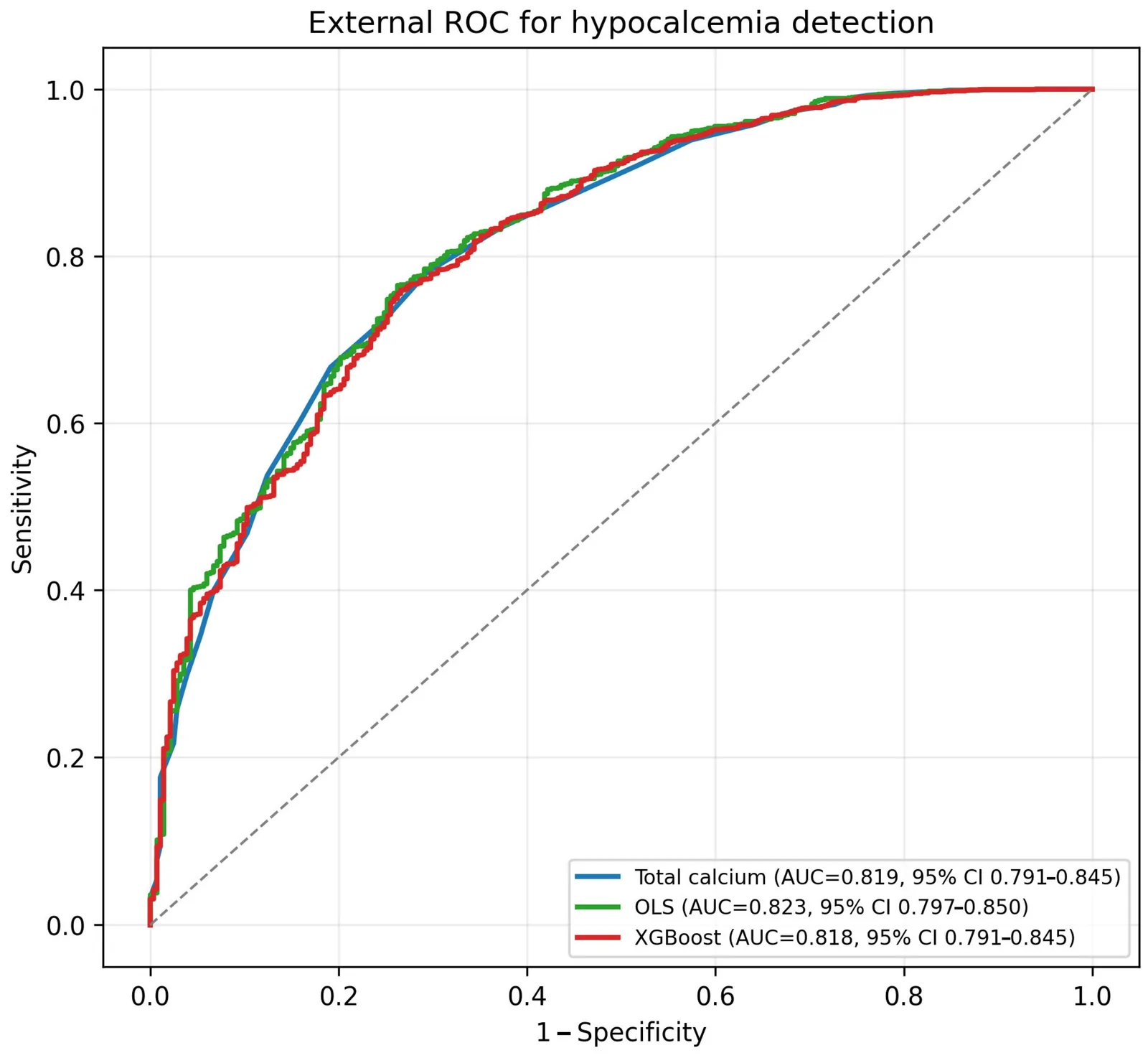
ROC curves of total calcium, OLS, and XGBoost for hypocalcemia detection in the external cohort.

For hypocalcemia detection in the external cohort, XGBoost and OLS retained a higher clinical sensitivity than unadjusted total calcium (98.5% and 95.8%, respectively, versus 78.1%), and discrimination was comparable across all three methods: AUC-ROC was 0.818 for XGBoost, 0.823 for OLS, and 0.819 for total calcium alone (Table 9 and Figure 7). Clinical specificity was lower for both models than for total calcium (27.0% for XGBoost, 37.2% for OLS, versus 70.6% for total calcium), reflecting the same sensitivity-specificity trade-off observed internally; in this cohort, XGBoost’s external specificity (27.0%) was similar to its internal specificity (26.0%), indicating that this trade-off did not markedly worsen when the model was applied outside the development center (Table 9). At these fixed thresholds, XGBoost produced 67 fewer false negatives and 29 additional false positives than OLS. Compared with the unadjusted total calcium, it produced 503 fewer false negatives and 123 additional false positives. These are descriptive differences in this external cohort and do not establish a clinical benefit.

Paired DeLong testing found no statistically significant differences among the three external ROC-AUCs after Holm adjustment (all adjusted *p* ≥ 0.192; all 95% confidence intervals for paired ΔAUC included zero; Supplementary Table S8). These findings do not establish equivalence or change fixed-threshold classification results.

The threshold-matched analysis changed the interpretation of the original high XGBoost sensitivity. With the development-derived specificity-matched threshold (<1.0872 mmol/L), external sensitivity and specificity were 77.6% and 70.2%, compared with 78.1% and 70.6% for unadjusted total calcium. The paired differences (XGBoost minus total calcium) were −0.005 for sensitivity (95% CI −0.015 to 0.005) and −0.004 for specificity (95% CI −0.038 to 0.032). With the development-derived sensitivity-matched threshold (<1.0853 mmol/L), external sensitivity was 75.9% and specificity was 73.4%; the paired differences were −0.022 (95% CI −0.032 to −0.012) and +0.028 (95% CI 0.000 to 0.062), respectively. Thus, after matching the operating point using development data, XGBoost did not show a clear incremental classification advantage over unadjusted total calcium in the external cohort (Supplementary Table S12).

Decision-curve analysis gave a similar message. In the external cohort, XGBoost, OLS, and unadjusted total calcium all had higher net benefit than treat-all over almost the full threshold-probability range. However, the difference between XGBoost and total calcium was small (mean ΔNB across the 0.05–0.95 grid = −0.0051; maximum ΔNB = 0.0157). At the prespecified threshold probabilities of 0.10, 0.20, 0.30, 0.40, and 0.50, all paired bootstrap 95% confidence intervals for XGBoost-minus-total-calcium net benefit included zero. Thus, the decision-curve analysis did not show clear incremental external decision value for XGBoost over unadjusted total calcium (Supplementary Table S13 and Figure S4).

In exploratory external subgroup analyses, the albumin groups contained 468, 836, and 1447 patients, and the eGFR groups contained 464, 546, and 1741 patients, respectively. XGBoost MAE ranged from 0.063 to 0.057 mmol/L across the albumin groups and from 0.062 to 0.057 mmol/L across the eGFR groups; the corresponding ROC-AUC ranges were 0.789–0.850 and 0.802–0.864. XGBoost sensitivity ranged from 96.3% to 99.4% across albumin groups; the unadjusted total calcium sensitivity was 95.0% for albumin < 25 g/L and 66.7% for albumin 30–35 g/L. Specificity in the albumin < 25 and eGFR < 30 groups was estimated from only 32 and 49 non-hypocalcemic patients, respectively (Supplementary Table S9). These comparisons were descriptive, and subgroup interactions were not formally tested.

In the external cohort, the mean absolute SHAP importance ranked total calcium first (mean |SHAP| = 0.0485; relative share 74.0%), followed by albumin (0.0094; 14.4%), total protein (0.0057; 8.7%), and eGFR (0.0019; 3.0%). This ordering was the same as the development-cohort XGBoost gain-importance ranking, although the percentage shares are not directly comparable because gain and mean absolute SHAP are different importance measures (Supplementary Table S11 and Figure S2).

Because hypocalcemia was frequent in both cohorts (82.5% in development and 89.7% in external validation), we also evaluated balanced accuracy and MCC using fixed-threshold binary classifications. For XGBoost, the balanced accuracy was 62.2% internally and 62.7% externally, while MCC was 0.398 and 0.389, respectively. In the external cohort, the balanced accuracy was 74.3% for total calcium and 66.5% for OLS, compared with 62.7% for XGBoost. These measures complement the accuracy and predictive values and make the low specificity at the selected threshold more apparent (Supplementary Table S3).

The three-class classification results for hypocalcemia, normocalcemia, and hypercalcemia in the external cohort are presented in Table 10. In the three-class comparison, the three-class accuracy was highest for XGBoost (90.9%), followed by OLS (89.6%), and total calcium (77.0%). As 89.7% of the external cohort was hypocalcemic, these accuracy values were strongly influenced by class prevalence and should not be interpreted independently of class-specific performance. OLS and XGBoost correctly classified only 31.1% and 19.1% of normocalcemic patients, respectively. These three-class accuracy values are not directly comparable with the binary accuracy for hypocalcemia versus non-hypocalcemia in Table 9, because Table 9 collapses normocalcemia and hypercalcemia into a single non-hypocalcemia class. Both models showed a pattern of overclassifying hypocalcemia among externally normocalcemic patients (68.1% for OLS and 79.4% for XGBoost misclassified as hypocalcemic, versus 31.5% for total calcium), consistent with the reduced clinical specificity reported in Table 9 and with a case mix containing proportionally fewer normocalcemic patients than the development cohort (Table 7). External three-class confusion-matrix heatmaps are provided in Supplementary Figure S3.

## 4. Discussion

This study evaluated the agreement and classification performance of unadjusted total calcium, adjusted formulas, and a machine learning-based approach against measured ionized calcium in patients with hypoalbuminemia. Total calcium and the Jain formula showed similar overall performance, with misclassification most notably reflected in the overestimation of hypocalcemia among normocalcemic individuals. Using nested cross-validation and hyperparameter tuning, the XGBoost model demonstrated the highest AUC-ROC and F1-score for hypocalcemia detection, although this was accompanied by an increased rate of false-positive classifications. The Simplified Payne formula demonstrated the weakest performance, primarily due to its markedly low clinical sensitivity for hypocalcemia, despite its relatively high clinical specificity.

In clinical practice, hypoalbuminemia is one of the most common causes of low total calcium levels, particularly in hospitalized patients. A decrease in albumin levels can lead to a decrease in the measured total calcium values, even though ionized calcium remains normal. Ionized calcium levels can be influenced by several factors, including acid–base status, citrate, heparin, and free fatty acids. These physiological relationships highlight the limitations of simple correction formulas based solely on total calcium and albumin levels [4,28,29,30]. Although ionized calcium is accepted as the reference standard for evaluating physiological calcium status, it is not routinely measured in all patients in many clinical laboratories. The main reasons for this are preanalytical requirements, specific sampling conditions, and measurement requirements generally associated with blood gas analysis. As a practical and cost-effective reflex testing approach, the use of unadjusted total calcium as an initial screening test may be considered, with measurement of ionized calcium reserved for confirmation in cases where the results fall outside the reference range or are inconsistent with the clinical context, particularly in patients with low eGFR, hypoalbuminemia, or critical illness [31].

Our findings are consistent with those of recent large-scale studies questioning the added clinical value of albumin-corrected calcium formulas compared with uncorrected total calcium. Numerous studies have highlighted the limitations of albumin-corrected calcium formulations [31]. Ladenson et al. [32] assessed 13 calcium adjustment formulas, including those of Orrell, Berry, and Payne, in a cohort of hospitalized patients and controls, and demonstrated that none outperformed unadjusted total calcium in correlating with ionized calcium. Compared to ionized calcium, albumin-corrected calcium has been reported to accurately classify calcium status in only 57–66% of cases [1,17,31,33,34]. A study by Desgagnés et al. showed that commonly used correction formulas do not consistently improve agreement with ionized calcium levels and may lead to systematic bias, particularly in patients with hypoalbuminemia [1]. The use of total calcium and albumin-corrected calcium formulas often leads to misclassification of patients’ calcium status. In particular, when clinical conditions such as altered albumin levels, renal function, or blood pH are present, the formulas cannot reliably estimate the calcium status. In patients with low albumin levels (<35 g/L) or abnormal blood pH, calcium corrected by the Payne formula tends to overestimate the calcium status in up to 80% of cases. Because the Clase, Jain, and Ridefelt formulas contain lower albumin coefficients than the Payne formula, they demonstrate a significant improvement (higher agreement rate) compared to the Payne formula in patients with low albumin levels. Consistent with the literature, we observed that total calcium alone performed better than most classical formulas in the hypoalbuminemia subgroup. Many formulas, particularly those based solely on albumin correction, such as the Simplified Payne formula, exhibited a clinically significant positive bias (overestimation). Consistent with previous reports, the Jain formula showed a modest improvement over total calcium and reduced the misclassification of hypocalcemia compared to the Payne formula. These findings support the growing view that fixed- or rule-based correction equations may not adequately reflect multifactorial determinants of calcium homeostasis in different clinical settings, including renal function and acid–base status [1].

Calcium homeostasis is regulated by the interaction of many factors, such as pH, electrolyte concentrations, protein composition, and kidney function [34]. Machine learning methods offer a more flexible approach than traditional correction formulas owing to their ability to capture nonlinear relationships and interactions between variables [2,5]. In our study, the inclusion of total protein and eGFR, alongside total calcium and albumin, aimed to capture complementary information about circulating protein status and renal handling of calcium without introducing the additional complexity and blood-gas-dependent nature of pH measurement. Thus, the model aimed to reflect the physiologically active calcium fraction more accurately in situations where ionized calcium measurements are not routinely available. In a general internal medicine cohort of 210 hospitalized patients, Alhenc-Gelas et al. [35] reported that the total calcium level achieved only 50% clinical sensitivity, despite a high clinical specificity of 95%, for the diagnosis of hypocalcemia; albumin- or protein-based corrections did not improve, and in some cases, the diagnostic accuracy was reduced. Similarly, in a surgical intensive care unit population, Byrnes et al. [36] demonstrated that corrected calcium (Simplified Payne) had limited clinical sensitivity (53%) and moderate clinical specificity (85%), with a tendency to underestimate true hypocalcemia and overestimate normocalcemia. In line with these findings, total calcium achieved a clinical sensitivity of 80.8% in our cohort, whereas the XGBoost model increased the clinical sensitivity to 98.5% using nested cross-validation out-of-fold predictions, substantially reducing false-negative classifications. Muller et al. [37] developed a random forest-based model to predict postoperative hypocalcemia following thyroidectomy, achieving strong discrimination with AUC values of 0.902 in the derivation cohort and 0.928 in the validation cohort. By selecting appropriate decision thresholds, the model achieved 92% clinical sensitivity and 90% clinical specificity, highlighting the ability of machine learning approaches to balance clinical sensitivity and specificity according to clinical priorities. Although this study focused on postoperative risk prediction rather than direct classification of calcium status, it supports the potential of ensemble tree-based methods to provide high diagnostic performance in calcium-related clinical applications [37]. In the present study, out-of-fold nested cross-validated XGBoost achieved the highest AUC-ROC (0.798) and highest clinical sensitivity (98.5%) for hypocalcemia detection; however, this was accompanied by a lower clinical specificity (26.0%) and a higher number of false-positive classifications. Differences in study design, outcome definition, and patient population preclude the direct comparison of performance metrics between studies. In patients undergoing chronic hemodialysis, a regression model using albumin, phosphate, total calcium, creatinine, and blood urea nitrogen provided better predictive performance (R^2^ = 0.81) for ionized calcium than unadjusted total calcium and conventional correction formulas (Payne, Clase, Jain, and Ferrari) [30]. Kumari et al. [5] demonstrated that an XGBoost model using only three variables (total calcium, total protein, and the degree of albumin reduction) provided a more accurate classification of calcium status than both unadjusted total calcium and conventional correction formulas, such as Payne, Jain, Orrell, and Berry; the Jain formula demonstrated the lowest deviation among conventional correction formulas (MAD = 0.986), although with a relatively weak correlation (r = 0.251) [5]. In line with these findings, the Jain formula showed the highest performance among the classical approaches evaluated in our study (R^2^ = 0.361), yielding an AUC-ROC of 0.791 and an F1-score of 86.5, and reduced the misclassification of hypocalcemia compared to the Simplified Payne formula. A recent study by Bancal et al. [2] applied a random forest-based model using total calcium, albumin, phosphate, and age to estimate ionized calcium and reported improved classification performance compared with unadjusted total calcium and conventional correction formulas, outperforming total calcium in identifying normocalcemia and hypercalcemia and achieving an overall classification accuracy of approximately 78% when ionized calcium was used as the reference standard. Unlike prior studies that evaluated model performance using data from their respective study centers, the present study additionally assessed the transportability of the developed models in an independent external hospital cohort. This evaluation revealed a reduction in explained variance and a small degree of miscalibration relative to internal out-of-fold estimates, while discrimination for hypocalcemia detection remained comparable to unadjusted total calcium, underscoring the importance of external evaluation before assuming the full transportability of internally validated performance. Our findings are broadly consistent with these observations and indicate that multivariate machine learning approaches may improve sensitivity and continuous prediction performance relative to unadjusted total calcium and conventional correction formulas, although external discrimination was similar to that of unadjusted total calcium. In our cohort, the out-of-fold XGBoost predictions achieved an AUC-ROC of 0.798 (95% CI 0.788–0.807) and a clinical sensitivity of 98.5% (95% CI 98.3–98.7%) for hypocalcemia detection; however, this gain in clinical sensitivity was accompanied by an increased rate of misclassification of normocalcemic cases as hypocalcemic, an expected trade-off under the marked class imbalance of the cohort (>80% hypocalcemia by the ionized reference), which underscores the importance of interpreting the AUC and F1-score together with clinical sensitivity, clinical specificity, and the corresponding confidence intervals. The threshold-matched external analysis, however, showed that the high sensitivity at the original XGBoost cutoff was largely an operating-point effect rather than evidence of better overall classification than unadjusted total calcium.

Recent literature further supports this direction. Kösem et al. [8], in a large cohort of 84,410 patients, reported improved continuous ionized-calcium prediction with support vector machine, random forest, and gradient boosting models compared with conventional correction formulas. Notably, however, their machine-learning models showed a pronounced sensitivity–specificity trade-off for hypocalcemia detection, with sensitivities of 98.7–98.9% but specificities of only 24.5–26.0%. This pattern closely resembles our internal findings for XGBoost (sensitivity, 98.5%; specificity, 26.0%), suggesting that the high sensitivity observed with machine-learning approaches should be interpreted together with the associated false-positive burden rather than as evidence of uniformly improved classification performance. This study highlighted external validation, pH incorporation, and model interpretability as priorities for future research [8]. The present study addresses these areas through independent external validation, pH sensitivity analysis, and external-cohort SHAP analysis; however, differences in population and study design preclude direct comparison of performance estimates.

The pH sensitivity analysis showed a small overall improvement in continuous ionized-calcium prediction when the recorded pH was added to the four routine serum predictors. In exploratory development-cohort strata, the original model tended to underestimate ionized calcium in acidemia and overestimate it in alkalemia; including pH brought the mean bias closer to zero in both groups. The absolute MAE reductions were small but numerically larger in the abnormal pH groups than in the normal pH group, without a formal test of effect modification. Lower continuous error did not translate into uniform fixed-threshold classification gains: in acidemia, adding pH reduced false positives but increased false negatives, while in alkalemia, the reverse trade-off was observed. These findings support the physiological relevance of pH but do not change the intended routine serum biochemistry use of the primary four-variable model. The pH-extended model and pH subgroup results have not been externally validated because the external cohort had no pH measurements.

Although total calcium accounted for 85.5% of the gain-based feature importance, ablation analysis indicated that albumin, total protein, and eGFR together provided a small improvement in continuous prediction beyond total calcium alone (Supplementary Tables S5–S7). This result does not establish the unique contribution of each predictor or additional clinical benefit. The total-calcium-only XGBoost regressor was distinct from the unadjusted total-calcium cutoff comparator.

Other potentially relevant variables, including phosphate, magnesium, citrate, inflammatory markers, PTH, and vitamin D levels, were not available in our dataset. Therefore, their possible contribution to ionized calcium prediction, particularly in specific clinical subgroups, could not be assessed. Future studies that include these variables may help to evaluate their additional predictive value.

In our laboratory, albumin-corrected calcium levels have been routinely reported using the Simplified Payne formula, which has been automatically calculated from the total calcium and albumin values in the LIS for many years. However, recent studies have questioned the reliability of this approach. Therefore, we evaluated the performance of classical correction formulas on our institutional dataset and observed significant discrepancies between albumin-corrected calcium and measured ionized calcium. Based on these findings, we developed machine learning models that were validated through nested cross-validation to improve the agreement between routine biochemical parameters and measured ionized calcium levels. As a transparent, computationally simple alternative, we derived an OLS equation for predicting ionized calcium from total calcium, albumin, total protein, and eGFR. Although it showed a slightly lower internal regression performance than XGBoost, this equation outperformed the classical fixed-coefficient formulas in the development cohort on the metrics reported here. Because the reported coefficients were obtained from a single fit of the full development cohort, their internal performance was estimated separately using nested cross validation. External validation on the same analytical platforms showed a moderate reduction in the explained variance and a small degree of miscalibration. Further multicenter validation, including centers using different analytical platforms and local recalibration, would nonetheless be advisable before the equation is adopted as a routine LIS-based implementation elsewhere [38,39]. Any future LIS integration would also require prospective verification, local validation and calibration, appropriate software and quality-control procedures, regulatory oversight, and ongoing monitoring of model performance, with periodic reassessment or updating as laboratory methods and patient population change. Beyond confirming previous findings, our internal validation demonstrated that data-driven multivariate approaches provided a performance advantage over total calcium alone in terms of explained variance and error metrics. In the external cohort, this advantage in explained variance persisted in relative terms, although the discrimination for hypocalcemia detection was comparable to that of unadjusted total calcium. When classical formulas were transformed to the ionized-calcium scale for comparison, the OLS and XGBoost models, evaluated using nested cross-validation out-of-fold predictions, showed higher explained variance, lower MAE and RMSE, and bias closer to zero than the fixed-coefficient formulas evaluated on the same ionized-calcium scale.

External application of the frozen XGBoost model and full-cohort OLS equation to an independent hospital cohort using the same analytical platforms provided an assessment of model performance outside the development cohort under similar analytical conditions. Regression R^2^ decreased from approximately 0.50 to 0.40 for XGBoost and from 0.46 to 0.36 for OLS; however, absolute prediction errors (MAE) were smaller than in development for both models, reflecting the narrower range of measured ionized calcium in this cohort. The narrower distribution of measured ionized calcium in the external cohort may have contributed to the lower R^2^ despite the smaller absolute prediction errors. Both models showed a small systematic positive bias of approximately 0.02 mmol/L, in contrast to the near-zero bias observed in development, together with calibration slopes below 1 (0.934 for XGBoost, 0.873 for OLS), indicating that the frozen predictions were modestly more dispersed than the observed ionized calcium values. Using an absolute SMD of 0.20 or greater as a descriptive threshold for at least a small standardized between-cohort difference, based on Cohen’s conventional effect-size criteria [40], the external cohort differed from the development cohort primarily in total protein and eGFR (|SMD| > 0.30), while hypocalcemia prevalence also showed a smaller but notable between-cohort difference (|SMD| = 0.209). The differences in total protein and eGFR may have contributed to the change in model performance, although their individual effects could not be determined from this comparison. The higher prevalence of hypocalcemia in the external cohort also affected the interpretation of the PPV, NPV, and overall accuracy. Therefore, these measures should be interpreted in relation to the case mix of each cohort. Discrimination for hypocalcemia detection was comparable across all three methods in the external cohort (AUC-ROC 0.818 for XGBoost, 0.823 for OLS, and 0.819 for total calcium), in contrast to the advantage of the machine learning models observed internally (AUC-ROC 0.798 versus 0.785 for total calcium). Notably, the AUC-ROC of unadjusted total calcium itself was higher in the external cohort than in the internal cohort, suggesting that at least part of this pattern reflects differences in case-mix separability between cohorts rather than a specific loss of model discrimination. The clinical specificity for XGBoost was similar internally and externally (26.0% versus 27.0%), indicating that this trade-off did not appreciably worsen outside the development center. These findings suggest that, when applied to a cohort assessed on the same analytical platforms as the development center, the models presented here retain a broadly similar performance profile, although the reduction in explained variance and the persistence of a sensitivity-specificity trade-off indicate that recalibration and further multicenter validation, discussed below, remain prudent before broader adoption. Consistent with the similar external ROC-AUC values, the development-derived threshold-matched analysis did not demonstrate clear incremental XGBoost classification value over unadjusted total calcium.

The exploratory albumin and eGFR subgroup summaries indicated numerical differences in the prediction error and fixed-threshold classification performance. Because hypocalcemia was common and some subgroups contained few non-hypocalcemic patients, the specificity estimates were imprecise. These findings do not establish the subgroup-specific clinical utility or causal effects of albumin or eGFR.

External SHAP analysis showed that total calcium remained the dominant contributor to the deployed XGBoost predictions, followed by albumin, total protein, and eGFR, reproducing the same feature ordering observed with development-cohort gain importance. Different relative percentages are expected because gain and mean absolute SHAP quantify importance differently. These SHAP attributions describe model behavior in the external cohort and should not be interpreted as causal effects of predictors of ionized calcium.

The paired external comparison showed a small improvement in continuous prediction with XGBoost over OLS, whereas the AUC values were similar for XGBoost, OLS, and unadjusted total calcium. At predefined thresholds, XGBoost missed fewer hypocalcemic patients but generated more false-positive results. In the external cohort, 206 of 282 patients without hypocalcemia were classified as hypocalcemic by XGBoost, 177 by OLS, and 83 by unadjusted total calcium. Compared with OLS, XGBoost produced 67 fewer false negatives and 29 more false positives; compared with total calcium, the differences were 503 and 123, respectively. If used to trigger confirmatory ionized calcium measurements, XGBoost would flag 2638 of the 2751 patients, compared to 2542 for OLS and 2012 for total calcium. This could increase the confirmatory testing and laboratory workload, although no testing costs or patient outcomes were measured. Because 89.7% of this selected cohort had hypocalcemia, these counts should not be used to estimate the testing burden in the general laboratory population. The clinical value of this sensitivity–specificity trade-off requires prospective evaluation. Sensitivity and specificity depend on the selected cutoff, while predictive values also depend on the prevalence of hypocalcemia. To address the effect of threshold choice, we also evaluated development-derived XGBoost thresholds matched to the sensitivity or specificity of total calcium; these thresholds were applied unchanged in the external cohort.

Formal paired DeLong comparisons did not establish an external ROC-AUC difference between XGBoost, OLS, and unadjusted total calcium levels after Holm adjustment (Supplementary Table S8). A non-significant comparison does not demonstrate equivalence; ROC discrimination is also distinct from sensitivity and specificity at fixed clinical thresholds.

At the original 1.15 mmol/L cutoff, the high sensitivity of XGBoost was obtained at the cost of low specificity. After threshold matching based only on development out-of-fold predictions, this apparent classification advantage did not persist in external validation. At the specificity-matched threshold, external sensitivity and specificity were almost the same as for unadjusted total calcium, and the sensitivity-matched analysis also did not show a clear overall advantage. These findings indicate that the original sensitivity gain should be interpreted mainly as an operating-point effect rather than superior discrimination. Any future LIS-based use would therefore require a prespecified clinical decision setting, prospective decision-impact evaluation, and assessment of costs and clinical outcomes before implementation.

The exploratory decision-curve analysis supported the same interpretation. Although XGBoost, OLS, and total calcium each showed higher net benefit than a treat-all strategy for the hypothetical decision to request confirmatory ionized-calcium measurement, net benefit was similar between XGBoost and unadjusted total calcium. Absolute differences were small, and the bootstrap confidence intervals for XGBoost-minus-total-calcium net benefit at the prespecified thresholds included zero. Taken together with the threshold-matched analysis, these results do not support a clear incremental clinical decision-value claim for XGBoost over unadjusted total calcium in the external cohort. The DCA remains exploratory because no harm ratio, testing-cost framework, or patient-outcome endpoint was prespecified.

These findings suggest a potentially specific rather than general clinical role for the models evaluated here. Because both XGBoost and the OLS equation exhibited high clinical sensitivity but low specificity, their most plausible future application may be as LIS-based triggers for confirmatory ionized calcium measurement rather than as standalone diagnostic tools or direct substitutes for measured ionized calcium. Such a strategy could be relevant in clinical settings where the consequences of missed hypocalcemia are considered more important than the burden of additional confirmatory testing. However, the high proportion of false-positive classifications observed both internally and externally indicates that implementation at the current thresholds could generate a substantial testing burden. Therefore, the clinical value of this approach would need to be established through prospective evaluation, threshold selection based on the intended clinical setting, and decision-curve or cost-effectiveness analysis. The transparent OLS equation showed a regression performance close to that of XGBoost internally and externally, as well as similar external discrimination for hypocalcemia, and may therefore represent a more practical candidate for future LIS-based evaluations.

This study had several strengths. The analysis was conducted on a large, real-world clinical dataset comprising 14,745 patients with hypoalbuminemia, a population in which calcium interpretation is particularly challenging. The model comparison used nested cross-validation with hyperparameter tuning, providing out-of-fold performance estimates with uncertainty intervals rather than a single train–test split. Classical formulas, linear regression approaches, and nonlinear machine learning models were compared within the same dataset, and the SHAP-based explainability analysis enhanced the interpretability of the selected model by quantifying the contribution of each variable to the prediction.

This study had some limitations. We performed an external validation in an independent hospital cohort using the same analyzer platforms, which showed a moderate reduction in regression performance, a small systematic positive bias, and calibration slopes below one relative to the development out-of-fold estimates. This pattern may partly reflect case-mix differences between centers, including a considerably higher prevalence of hypocalcemia and a narrower distribution of measured ionized calcium in the external cohort. Although the same analyzer models were used, residual between-laboratory analytical and pre-analytical differences cannot be excluded. No recalibration or threshold re-optimization was performed for this external evaluation. Our external validation in one additional hospital assessed performance under comparable analytical conditions, but it did not establish multicenter transportability or generalizability to laboratories using different analyzer platforms, preanalytical workflows, or patient case mixes. Further independent validation across diverse centers, with an assessment of calibration across settings, is required before broader clinical implementation. In addition, decision-curve analysis was performed only as an exploratory assessment of a hypothetical confirmatory-testing strategy. No harm ratio for missed hypocalcemia versus unnecessary ionized-calcium testing was prespecified, and no cost or clinical-outcome data were available. The original prediction models remained continuous regressors; development-derived logistic mappings were used only to obtain probabilities for the DCA and were not used to alter the model predictions. Serum creatinine and pH were not included in the final predictor set: creatinine was excluded because of its strong inverse correlation with eGFR, and pH was excluded to avoid dependence on blood-gas sampling beyond the ionized calcium measurement itself, which may limit model performance in patients with marked acid–base disorders. Age and sex were not used as model inputs in either cohort, although their distributions were comparable between the development and external cohorts. This reflects the predictive-validation scope of the present study; prospective decision-impact studies with a prespecified clinical action and threshold-probability framework would be required before clinical implementation. Accordingly, external validation should be interpreted as evidence of predictive transportability under comparable analytical conditions, not as evidence that the models are ready for routine clinical implementation. The matched thresholds were derived from development out-of-fold predictions and transported unchanged; their external results further show that threshold-specific performance may vary across case mixes even when overall discrimination is similar. Both cohorts also required an available measured ionized-calcium result and therefore represent patients already selected for ionized-calcium testing. This selection may have contributed to the high prevalence of hypocalcemia (82.5% in development and 89.7% in external validation) and limits direct extrapolation of PPV, NPV, testing burden, and decision-curve findings to the general hypoalbuminemic laboratory population. Formal fairness analyses were not performed. Potential differences in model performance across demographic and clinical subgroups should be evaluated in future studies before clinical implementation. The model has not yet been deployed in a clinical information system or laboratory workflow. User interaction, handling of missing input variables, and workflow integration were therefore not evaluated and should be addressed in prospective implementation studies.

## 5. Conclusions

In conclusion, this study demonstrated that traditional albumin-corrected calcium formulas provide only limited improvement over unadjusted total calcium levels, and may introduce a systematic positive bias, particularly in patients with hypoalbuminemia. XGBoost and OLS provided continuous estimates of ionized calcium and showed lower prediction error than the fixed formulas in development, but external discrimination for hypocalcemia was similar to unadjusted total calcium. At the original 1.15 mmol/L cutoff, XGBoost had very high sensitivity with low specificity. When alternative XGBoost thresholds were selected only from development data to match total-calcium specificity or sensitivity and then applied unchanged to the external cohort, no clear incremental classification value was observed. Exploratory decision-curve analysis also showed no clear incremental net-benefit advantage for XGBoost over unadjusted total calcium. The models should therefore not be considered substitutes for measured ionized calcium. Further multicenter validation and prospective decision-impact evaluation are needed before clinical use.

## Figures and Tables

**Table 1:** Descriptive characteristics of the hypoalbuminemia analytic cohort (*n* = 14,745).

Variable	Mean ± SD	Median (IQR)
Total calcium, mmol/L	2.09 ± 0.23	2.08 (1.96–2.20)
Albumin, g/L	29.0 ± 4.27	29.7 (26.0–32.5)
Total protein, g/L	58.9 ± 9.05	58.7 (53.2–64.3)
eGFR, mL/min/1.73 m^2^	60.5 ± 38.7	57.3 (25.5–90.7)
Ionized calcium, mmol/L	1.06 ± 0.13	1.06 (0.99–1.12)

SD, standard deviation; IQR, interquartile range; eGFR, estimated glomerular filtration rate.

**Table 2:** Nested cross-validated machine learning performance for predicting measured ionized calcium in hypoalbuminemia.

Model	R^2^ OOF (95% CI)	MAE OOF (95% CI)	RMSE OOF (95% CI)	Bias OOF	Bias OOF (%)
XGBoost	0.500 (0.477–0.522)	0.066 (0.065–0.067)	0.092 (0.090–0.095)	−0.00052	−0.049
Random Forest	0.496 (0.474–0.517)	0.066 (0.065–0.067)	0.093 (0.091–0.095)	−0.00032	−0.030
Elastic Net	0.458 (0.437–0.476)	0.068 (0.067–0.069)	0.096 (0.094–0.099)	−0.00048	−0.045
OLS	0.458 (0.437–0.476)	0.068 (0.067–0.069)	0.096 (0.094–0.099)	−0.00048	−0.045
LASSO	0.457 (0.437–0.476)	0.068 (0.067–0.069)	0.096 (0.094–0.099)	−0.00048	−0.045
Ridge	0.458 (0.437–0.476)	0.068 (0.067–0.069)	0.096 (0.094–0.099)	−0.00048	−0.045

The units for MAE, RMSE, and bias are mmol/L. OOF, out-of-fold; CI, confidence interval; MAE, mean absolute error; RMSE, root mean square error.

**Table 3:** Performance of published calcium correction and ionized-calcium estimation equations and unadjusted total calcium in hypoalbuminemia.

Formula	*n*	R^2^	MAE	RMSE	Bias	Bias (%)
Jain	14,745	0.361	0.079	0.105	0.046	5.17
TC	14,745	0.351	0.079	0.105	0.042	4.71
Ridefelt	14,745	0.210	0.092	0.116	0.069	7.36
Orrell	14,745	0.097	0.100	0.124	0.081	8.54
Clase	14,745	0.009	0.107	0.130	0.090	9.38
James	14,745	−0.097	0.114	0.137	0.100	10.33
Pekar	14,741	−0.158	0.118	0.141	0.103	10.60
Antonio (A)	14,745	−0.359	0.131	0.152	0.116	12.08
Antonio (C)	14,745	−0.371	0.132	0.153	0.117	12.22
Parfitt	14,745	−0.531	0.138	0.162	0.125	12.77
Simplified Payne	14,745	−0.666	0.148	0.169	0.140	14.16
Dent	14,745	−0.793	0.151	0.175	0.140	14.23
Walker	14,745	−0.926	0.161	0.181	0.154	15.55
Thode	14,745	−1.091	0.168	0.189	0.162	16.22
Payne	14,745	−1.118	0.170	0.190	0.164	16.50
Berry	14,745	−2.165	0.214	0.233	0.212	21.10
Antonio (B)	14,745	−7.773	0.263	0.387	−0.079	−6.79

Pekar *n* = 14,741 because the blood pH required by this formula was missing in four patients; all other formulas were evaluated in the full cohort (*n* = 14,745). The units for MAE, RMSE, and bias are mmol/L. MAE, mean absolute error; RMSE, root mean square error; TC, total calcium.

**Table 4:** Comparison of total calcium, correction formulas, and XGBoost for calcium classification.

Reference Classification According to Ionized Calcium	Predicted Classification			
	Hypocalcemia	Normocalcemia	Hypercalcemia	Total
**Classification by XGBoost**				
Hypocalcemia	11,989 (98.5)	180 (1.50)	2 (0.00)	12,171 (82.5)
Normocalcemia	1843 (82.1)	373 (16.6)	29 (1.30)	2245 (15.2)
Hypercalcemia	63 (19.1)	80 (24.3)	186 (56.5)	329 (2.20)
Total	13,895 (94.2)	633 (4.30)	217 (1.50)	14,745 (100)
Observed Agreement (overall accuracy)				85.1%
**Classification by total calcium**				
Hypocalcemia	9834 (80.8)	2333 (19.2)	4 (0.00)	12,171 (82.5)
Normocalcemia	993 (44.2)	1206 (53.7)	46 (2.00)	2245 (15.2)
Hypercalcemia	17 (5.20)	110 (33.4)	202 (61.4)	329 (2.20)
Total	10,844 (73.5)	3649 (24.7)	252 (1.70)	14,745 (100)
Observed Agreement (overall accuracy)				76.2%
**Classification by adjusted calcium using the Jain formula**				
Hypocalcemia	10,066 (82.7)	2102 (17.3)	3 (0.00)	12,171 (82.5)
Normocalcemia	1028 (45.8)	1165 (51.9)	52 (2.30)	2245 (15.2)
Hypercalcemia	20 (6.10)	99 (30.1)	210 (63.8)	329 (2.20)
Total	11,114 (75.4)	3366 (22.8)	265 (1.80)	14,745 (100)
Observed Agreement (overall accuracy)				77.6%
**Classification by adjusted calcium using the Simplified Payne formula**				
Hypocalcemia	3490 (28.7)	8620 (70.8)	61 (0.50)	12,171 (82.5)
Normocalcemia	137 (6.10)	1846 (82.2)	262 (11.7)	2245 (15.2)
Hypercalcemia	1 (0.30)	70 (21.3)	258 (78.4)	329 (2.20)
Total	3628 (24.6)	10,536 (71.4)	581 (3.94)	14,745 (100)
Observed Agreement (overall accuracy)				37.9%

Rows indicate the reference classification according to the measured ionized calcium, and columns indicate the classification predicted by each method. Total-calcium classification used the original code-defined limits of 2.1956 and 2.6447 mmol/L (hypocalcemia < 2.1956; hypercalcemia > 2.6447); the routinely reported clinical reference interval was 2.20–2.65 mmol/L.

**Table 5:** Diagnostic performance of total calcium, correction formulas, and XGBoost for hypocalcemia detection in patients with hypoalbuminemia.

Metric	Simplified Payne	Total Calcium	Jain Formula	XGBoost (OOF)
TP	3490	9834	10,066	11,989
FP	138	1010	1048	1906
FN	8681	2337	2105	182
TN	2436	1564	1526	668
Clinical Sensitivity (%)	28.7 (27.9–29.5)	80.8 (80.1–81.5)	82.7 (82.0–83.4)	98.5 (98.3–98.7)
Clinical Specificity (%)	94.6 (93.7–95.4)	60.8 (58.9–62.6)	59.3 (57.4–61.2)	26.0 (24.3–27.7)
PPV (%)	96.2 (95.5–96.8)	90.7 (90.1–91.2)	90.6 (90.0–91.1)	86.3 (85.7–86.8)
NPV (%)	21.9 (21.2–22.7)	40.1 (38.6–41.6)	42.0 (40.4–43.6)	78.6 (75.7–81.2)
AUC-ROC	0.784 (0.773–0.793)	0.785 (0.774–0.794)	0.791 (0.780–0.800)	0.798 (0.788–0.807)
F1-Score	44.2	85.5	86.5	92.0
Binary accuracy (%)	40.2	77.3	78.6	85.8

The XGBoost metrics use nested cross-validation out-of-fold predictions. TP, true positive; FP, false positive; FN, false negative; TN, true negative; PPV, positive predictive value; NPV, negative predictive value; AUC-ROC, area under the receiver operating characteristic curve. Total-calcium classification used the original code-defined limits of 2.1956 and 2.6447 mmol/L (hypocalcemia < 2.1956; hypercalcemia > 2.6447); the routinely reported clinical reference interval was 2.20–2.65 mmol/L.

**Table 6:** External cohort characteristics.

Variable	Mean ± SD	Median (IQR)
Total calcium, mmol/L	2.08 ± 0.21	2.10 (1.97–2.20)
Albumin, g/L	29.3 ± 4.45	30.3 (26.6–32.9)
Total protein, g/L	56.3 ± 8.18	56.4 (51.5–61.1)
eGFR, mL/min/1.73 m^2^	72.9 ± 36.4	78.9 (43.9–99.9)
Ionized calcium, mmol/L	1.04 ± 0.11	1.05 (0.99–1.10)

SD, standard deviation; IQR, interquartile range; eGFR, estimated glomerular filtration rate.

**Table 7:** Comparison of Characteristics Between the Development and External Validation Cohorts.

Variable	Development (*n* = 14,745)	External (*n* = 2751)	SMD
Total calcium, mmol/L	2.09 ± 0.23	2.08 ± 0.21	0.023
Albumin, g/L	29.0 ± 4.27	29.3 ± 4.45	−0.075
Total protein, g/L	58.9 ± 9.05	56.3 ± 8.18	0.309
eGFR, mL/min/1.73 m^2^	60.5 ± 38.7	72.9 ± 36.4	−0.333
Ionized calcium, mmol/L	1.06 ± 0.13	1.04 ± 0.11	0.134
Hypocalcemia, *n* (%)	12,171 (82.5)	2469 (89.7)	−0.209
Normocalcemia, *n* (%)	2245 (15.2)	257 (9.3)	0.180
Hypercalcemia, *n* (%)	329 (2.2)	25 (0.9)	0.107

eGFR, estimated glomerular filtration rate; SMD, standardized mean difference (development minus external).

**Table 8:** External validation regression and calibration performance.

Model	External R^2^ (95% CI)	External MAE (95% CI)	External RMSE (95% CI)	External Bias, mmol/L (95% CI)	Calibration Slope (95% CI)	Calibration Intercept (95% CI)
XGBoost	0.397 (0.326–0.460)	0.059 (0.057–0.061)	0.082 (0.078–0.086)	0.018 (0.015–0.021)	0.934 (0.894–0.975)	0.052 (0.009–0.095)
OLS	0.363 (0.294–0.420)	0.060 (0.058–0.063)	0.084 (0.080–0.088)	0.016 (0.013–0.019)	0.873 (0.832–0.913)	0.119 (0.076–0.162)

Bias is defined as mean (predicted − observed); a positive value indicates a small systematic overestimation of measured ionized calcium. A calibration slope below 1 indicated that the frozen predictions were more dispersed than the observed ionized calcium values. The calibration intercept, mean bias, and slope were therefore interpreted jointly to characterize the mild-to-moderate calibration deviation observed.

**Table 9:** Diagnostic performance of total calcium, OLS, and XGBoost for hypocalcemia detection in the external cohort.

Metric	Total Calcium	OLS	XGBoost
TP	1929	2365	2432
FP	83	177	206
FN	540	104	37
TN	199	105	76
Clinical Sensitivity (%)	78.1 (76.5–79.7)	95.8 (94.9–96.5)	98.5 (97.9–98.9)
Clinical Specificity (%)	70.6 (65.0–75.6)	37.2 (31.8–43.0)	27.0 (22.1–32.4)
PPV (%)	95.9 (94.9–96.7)	93.0 (92.0–94.0)	92.2 (91.1–93.2)
NPV (%)	26.9 (23.9–30.2)	50.2 (43.5–57.0)	67.3 (58.2–75.2)
AUC-ROC	0.819 (0.791–0.845)	0.823 (0.797–0.850)	0.818 (0.791–0.845)
F1-Score	86.1	94.4	95.2
Binary accuracy *	77.4	89.8	91.2

TP, true positive; FP, false positive; FN, false negative; TN, true negative; PPV, positive predictive value; NPV, negative predictive value; AUC-ROC, area under the receiver operating characteristic curve. Positive class: hypocalcemia (measured ionized calcium < 1.15 mmol/L). * Binary accuracy for hypocalcemia versus non-hypocalcemia is the proportion of patients correctly classified as hypocalcemic or non-hypocalcemic and is not identical to the three-class accuracy in Table 10. Total-calcium classification used the original code-defined limits of 2.1956 and 2.6447 mmol/L (hypocalcemia < 2.1956; hypercalcemia > 2.6447); the routinely reported clinical reference interval was 2.20–2.65 mmol/L.

**Table 10:** Comparison of total calcium, OLS, and XGBoost for three-class calcium classification in the external cohort.

*n* = 2751	Predicted Classification			
Reference Classification According to Measured Ionized Calcium	Hypocalcemia	Normocalcemia	Hypercalcemia	Total
**Classification by total calcium**				
**Hypocalcemia**	1929 (78.1)	539 (21.8)	1 (0.04)	2469 (89.7)
**Normocalcemia**	81 (31.5)	169 (65.8)	7 (2.72)	257 (9.30)
**Hypercalcemia**	2 (8.00)	4 (16.0)	19 (76.0)	25 (0.90)
**Total**	2012 (73.1)	712 (25.9)	27 (0.98)	2751 (100)
Three-class accuracy				77.0%
**Classification by OLS**				
**Hypocalcemia**	2365 (95.8)	103 (4.17)	1 (0.04)	2469 (89.7)
**Normocalcemia**	175 (68.1)	80 (31.1)	2 (0.78)	257 (9.30)
**Hypercalcemia**	2 (8.00)	4 (16.0)	19 (76.0)	25 (0.90)
**Total**	2542 (92.4)	187 (6.80)	22 (0.80)	2751 (100)
Three-class accuracy				89.6%
**Classification by XGBoost**				
**Hypocalcemia**	2432 (98.5)	36 (1.46)	1 (0.04)	2469 (89.7)
**Normocalcemia**	204 (79.4)	49 (19.1)	4 (1.56)	257 (9.30)
**Hypercalcemia**	2 (8.00)	3 (12.0)	20 (80.0)	25 (0.90)
**Total**	2638 (95.9)	88 (3.20)	25 (0.91)	2751 (100)
Three-class accuracy				90.9%

Rows indicate the reference classification according to the measured ionized calcium, and columns indicate the classification predicted by each method. Three-class accuracy was reported. Row percentages are shown in parentheses. Hypercalcemia was uncommon in the external cohort (*n* = 25, 0.9%); three-class results for this category should be interpreted cautiously given the small sample size. Three-class accuracy is the proportion of patients correctly assigned to hypocalcemia, normocalcemia, or hypercalcemia and differs from the binary hypocalcemia-versus-non-hypocalcemia accuracy in Table 9. Total-calcium classification used the original code-defined limits of 2.1956 and 2.6447 mmol/L (hypocalcemia < 2.1956; hypercalcemia > 2.6447); the routinely reported clinical reference interval was 2.20–2.65 mmol/L.

## Data Availability

The original contributions presented in this study are included in the article. Further inquiries can be directed to the corresponding author. The analytical code used for model development, validation, and supplementary analyses is available from the corresponding author upon reasonable request. The final trained XGBoost model object and the code required to generate predictions are available from the corresponding author upon reasonable request.

## Supplementary Materials

The following supporting information can be downloaded at: https://www.mdpi.com/article/10.3390/diagnostics16203381/s1, Supplementary Methods S1: Predictor ablation analysis; Supplementary Methods S2: Class-imbalance-aware binary classification; Supplementary Methods S3: Paired DeLong comparison of external ROC-AUC values; Supplementary Methods S4: Exploratory external subgroup performance; Supplementary Methods S5: Exploratory development-cohort pH subgroup analysis; Supplementary Methods S6: Selection of the final XGBoost hyperparameter configuration; Supplementary Methods S7: External-cohort SHAP explainability analysis; Supplementary Methods S8: Threshold-matched comparison of XGBoost and unadjusted total calcium; Supplementary Methods S9: Decision-curve analysis; Figure S1: Confusion matrices for the evaluated calcium classification methods in the development cohort; Figure S2: SHAP summary plot for the saved final XGBoost model in the independent external validation cohort; Figure S3: Three-class confusion-matrix heatmaps for total calcium, OLS, and XGBoost in the external validation cohort; Figure S4: Decision-curve analysis for a hypothetical confirmatory ionized-calcium testing strategy in the external cohort; Table S1: Adjusted total-calcium formulas and ionized-calcium–estimating equations evaluated in this study; Table S2: Frequency of complete XGBoost hyperparameter combinations selected across the 10 outer nested cross-validation folds; Table S3: Binary hypocalcemia classification metrics accounting for class imbalance in development and external validation cohorts; Table S4: Development-cohort out-of-fold prediction performance across pH categories (*n* = 14,741); Table S5: Out-of-fold regression performance of XGBoost predictor-ablation models in the development cohort (*n* = 14,745); Table S6: Paired differences in out-of-fold performance between predictor-ablation models; Table S7: Hypocalcemia classification by the four XGBoost ablation models at the fixed ionized-calcium threshold of <1.15 mmol/L (development cohort; *n* = 14,745); Table S8: External ROC-AUC estimates and paired DeLong comparisons (*n* = 2751); Table S9: External subgroup performance according to observed albumin and eGFR in the independent validation cohort (*n* = 2751); Table S10: Development-derived winsorization bounds applied to the external validation cohort, with the number of external values below, above, and winsorized at each bound; Table S11: SHAP-based feature importance of the final XGBoost model in the external validation cohort (*n* = 2751); Table S12: External threshold-matched comparison of XGBoost and unadjusted total calcium (*n* = 2751); Table S13: Decision-curve analysis in the independent external validation cohort (*n* = 2751).
