# Supplementary material for: Development and External Validation of Machine Learning Models for Ionized Calcium Estimation in Hypoalbuminemia
Source: Diagnostics (Basel). 2026 Oct 17;16(20):3381. doi: 10.3390/diagnostics16203381 (PMC13703762; doi:10.3390/diagnostics16203381)
Supplement: Supplementary file 1 [file diagnostics-16-03381-s001.zip › diagnostics-4555728-supplementary.pdf]

Supplementary Methods

S1. Predictor ablation analysis

In the development cohort (n = 14,745), four XGBoost regressors were compared using total calcium (TC) alone (A); TC and albumin (B); TC, albumin, and total protein (C); and TC, albumin, total protein, and eGFR (D, the primary model). The same observations and measured ionized-calcium outcome were used for all models. All models used identical 10 outer and five inner cross-validation splits (random seed 42), the original hyperparameter grid, and inner-fold tuning by negative RMSE. The original four-variable model remained the primary model; ablation models were not assessed in the external validation cohort.

Following the original model-development procedure, predictor winsorization bounds (Q1 − 3 × IQR, Q3 + 3 × IQR) and the training-target upper 99.7th percentile cap were estimated from each outer training fold and applied before the inner grid search. These bounds were not re-estimated separately within each inner training split. Outer test outcomes were not modified, and all reported development performance estimates were calculated from out-of-fold predictions against the original measured ionized-calcium values. Paired differences used 2,000 patient-level bootstrap resamples (seed 42), using identical patient indices for the compared models and 95% percentile confidence intervals.

Hypocalcemia was defined by measured or predicted ionized calcium <1.15 mmol/L, with no threshold optimization. A TC-only XGBoost regression model predicting ionized calcium should not be confused with the separate unadjusted TC clinical-threshold comparator evaluated in the main manuscript. The sequential differences depend on the stated feature-addition order and should not be interpreted as unique causal contributions.

S2. Class-imbalance-aware binary classification

Balanced accuracy was calculated as (sensitivity + specificity)/2. MCC was computed as (TP × TN − FP × FN)/√[(TP + FP)(TP + FN)(TN + FP)(TN + FN)]. Both measures were calculated directly from the existing binary confusion matrices, with hypocalcemia (measured ionized calcium <1.15 mmol/L) as the positive class. The original fixed thresholds were retained; the total-calcium comparator used the code-defined hypocalcemia cutoff <2.1956 mmol/L. The analysis did not involve model refitting, class resampling, or threshold optimization. Binary accuracy refers to hypocalcemia versus non-hypocalcemia, not the three-class classification in the main article.

S3. Paired DeLong comparison of external ROC-AUC values

Using the original external continuous predictions for the same 2,751 patients (2,469 with measured ionized calcium <1.15 mmol/L; 282 without), ROC ranking scores were the negatives of predicted ionized calcium (XGBoost and OLS) or measured total calcium. No model was retrained and no classification threshold was applied to the continuous ROC scores. Correlated AUCs and their 95% confidence intervals were estimated using the paired DeLong midrank method, which accounts for within-patient correlation and ties. The three two-sided paired p-values were adjusted using the Holm procedure ($\alpha = 0.05$). Confidence intervals for paired ΔAUC were unadjusted 95% DeLong intervals; adjusted p-values are reported separately. The original total-calcium and scale-aligned total-calcium scores gave identical ROC-AUC values.

S4. Exploratory external subgroup performance

The frozen external validation predictions (n = 2,751) were grouped by original observed albumin (<25, 25–<30, 30–<35 g/L) and eGFR (<30, 30–<60, ≥60 mL/min/1.73 m^2). All patients were included exactly once in each partition. XGBoost and OLS regression metrics were calculated on the ionized-calcium scale; unadjusted total calcium was evaluated for classification and ROC-AUC only. Reference hypocalcemia and predicted model hypocalcemia used <1.15 mmol/L; unadjusted total calcium used the original exact code threshold <2.1956 mmol/L. Subgroup membership and thresholds were not re-optimized and models were not retrained. 95% confidence intervals used 2,000 patient-level bootstrap resamples (seed 42; stratified for ROC-AUC), except sensitivity and specificity, for which Wilson intervals were used. One eGFR value above 200 mL/min/1.73 m^2 was audited and retained.

S5. Exploratory development-cohort pH subgroup analysis

The pH-complete development-cohort out-of-fold predictions from the earlier sensitivity analysis (n = 14,741; original four-variable XGBoost model A and pH-extended model B) were grouped by observed pH: acidemia (<7.35), normal pH (7.35–7.45, inclusive), and alkalemia (>7.45). No models were retrained. The original measured ionized-calcium outcome and fixed classification threshold (<1.15 mmol/L) were retained. Paired model differences used 2,000 patient-level bootstrap resamples (seed 42), with identical resampled observations for both models. Confidence intervals for sensitivity and specificity used the Wilson method, and ROC-AUC used negative continuous predictions. These exploratory results are not external validation: pH was unavailable in the external cohort. Arterial/venous sampling origin could not be distinguished in the available analysis fields. Extreme recorded pH observations were retained and flagged for audit.

S6. Selection of the final XGBoost hyperparameter configuration

Within each outer training fold, XGBoost was tuned by five-fold inner grid search using negative RMSE. The complete best-performing hyperparameter combination for each outer fold was counted once. The combination appearing most frequently across the 10 outer folds (3/10) was used to fit one final XGBoost model on the full development cohort (N = 14,745); individual parameter-wise modes were not combined. Selection frequency was a practical aggregation rule and does not demonstrate statistical optimality. The external cohort was not used for hyperparameter selection or refitting.

S7. External-cohort SHAP explainability analysis

SHAP values were calculated for all 2,751 observations in the independent external validation cohort using TreeExplainer applied to the saved final XGBoost model loaded by the external-validation pipeline. The model was not retrained or retuned. Development-derived 3×IQR winsorization bounds stored with the model artifact were applied unchanged before prediction and SHAP calculation. Global feature importance was summarized as the mean absolute SHAP value across patients, with relative shares obtained by normalizing the four mean absolute SHAP values to sum to 100%. These attribution measures describe model prediction contributions and are not causal effects.

S8. Threshold-matched comparison of XGBoost and unadjusted total calcium

To separate operating-point effects from overall discrimination, two alternative XGBoost hypocalcemia thresholds were selected using development-cohort out-of-fold predictions only. The specificity-matched threshold (<1.0872 mmol/L) was chosen to match the development specificity of unadjusted total calcium at <2.1956 mmol/L (60.8%). The sensitivity-matched threshold (<1.0853 mmol/L) was chosen to match the corresponding development sensitivity of total calcium (80.8%). Both XGBoost thresholds were frozen before external evaluation and were applied unchanged to the independent external cohort (n = 2,751). No external outcome was used for threshold selection. Paired XGBoost-minus-total-calcium differences were estimated with 2,000 patient-level bootstrap resamples (seed 42) and 95% percentile confidence intervals.

S9. Decision-curve analysis

Because XGBoost, OLS, and total calcium provide continuous calcium values rather than event probabilities, separate univariable logistic mappings were fitted in the development cohort. Hypocalcemia (measured ionized calcium <1.15 mmol/L) was modeled from the negative continuous calcium score so that lower calcium corresponded to higher estimated risk. Development out-of-fold predictions were used for XGBoost and OLS, while observed total calcium was used for the total-calcium mapping. These equations were frozen and applied unchanged to the external cohort; no external recalibration or threshold optimization was performed. Decision-curve analysis considered a hypothetical action of requesting confirmatory ionized-calcium measurement and compared XGBoost, OLS, unadjusted total calcium, treat-all, and treat-none over threshold probabilities 0.05–0.95 (step 0.01). Paired patient-level bootstrap resampling (2,000 resamples; seed 42) provided 95% percentile confidence intervals for net-benefit differences at threshold probabilities 0.10–0.50. No harm ratio for missed hypocalcemia versus unnecessary testing was prespecified, so the analysis was exploratory.

Supplementary Figures

Figure S1. Confusion matrices for the evaluated calcium classification methods in the development cohort

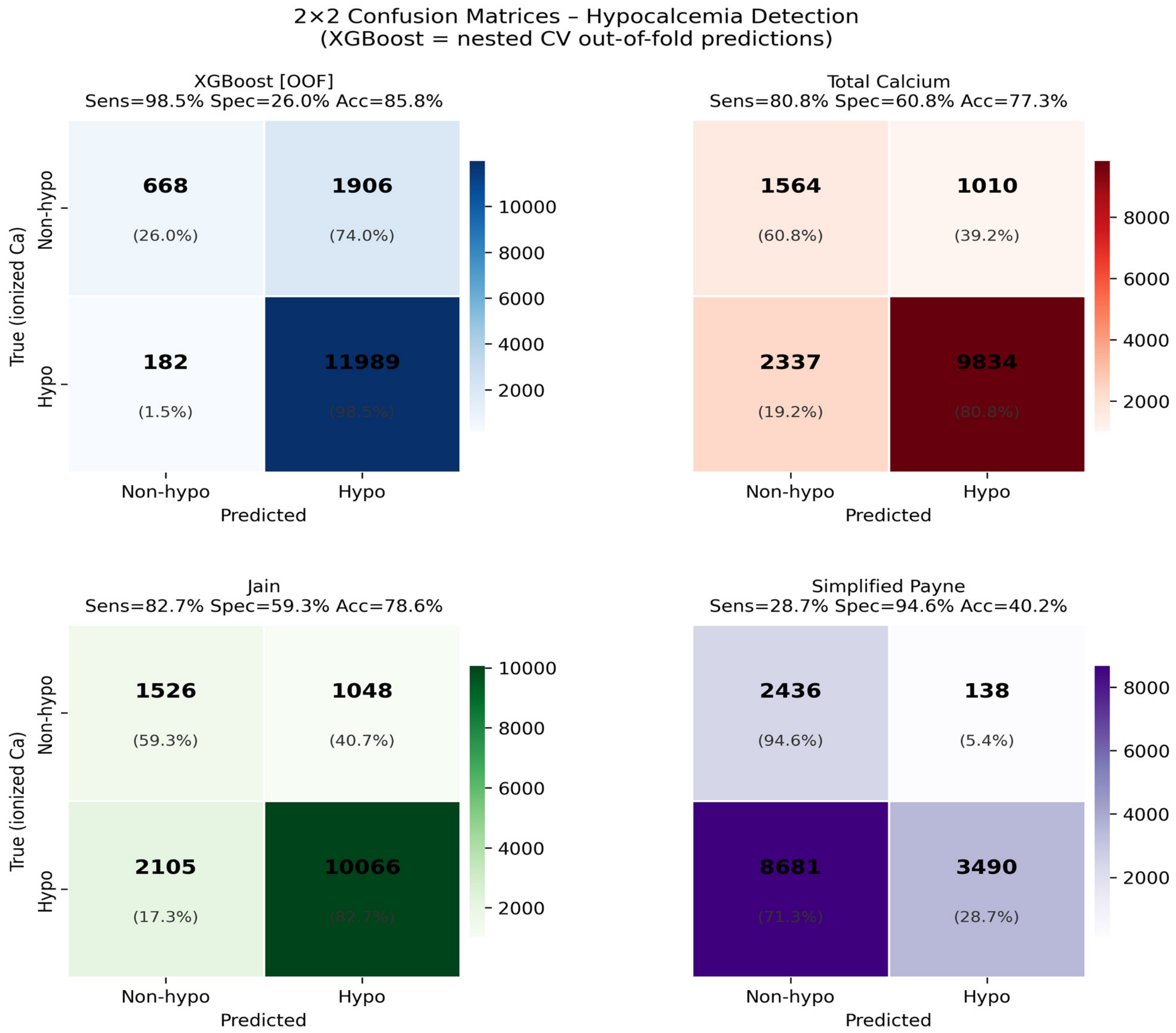

XGBoost results are based on nested cross-validation out-of-fold predictions.

Figure S2. SHAP summary plot for the saved final XGBoost model in the independent external validation cohort

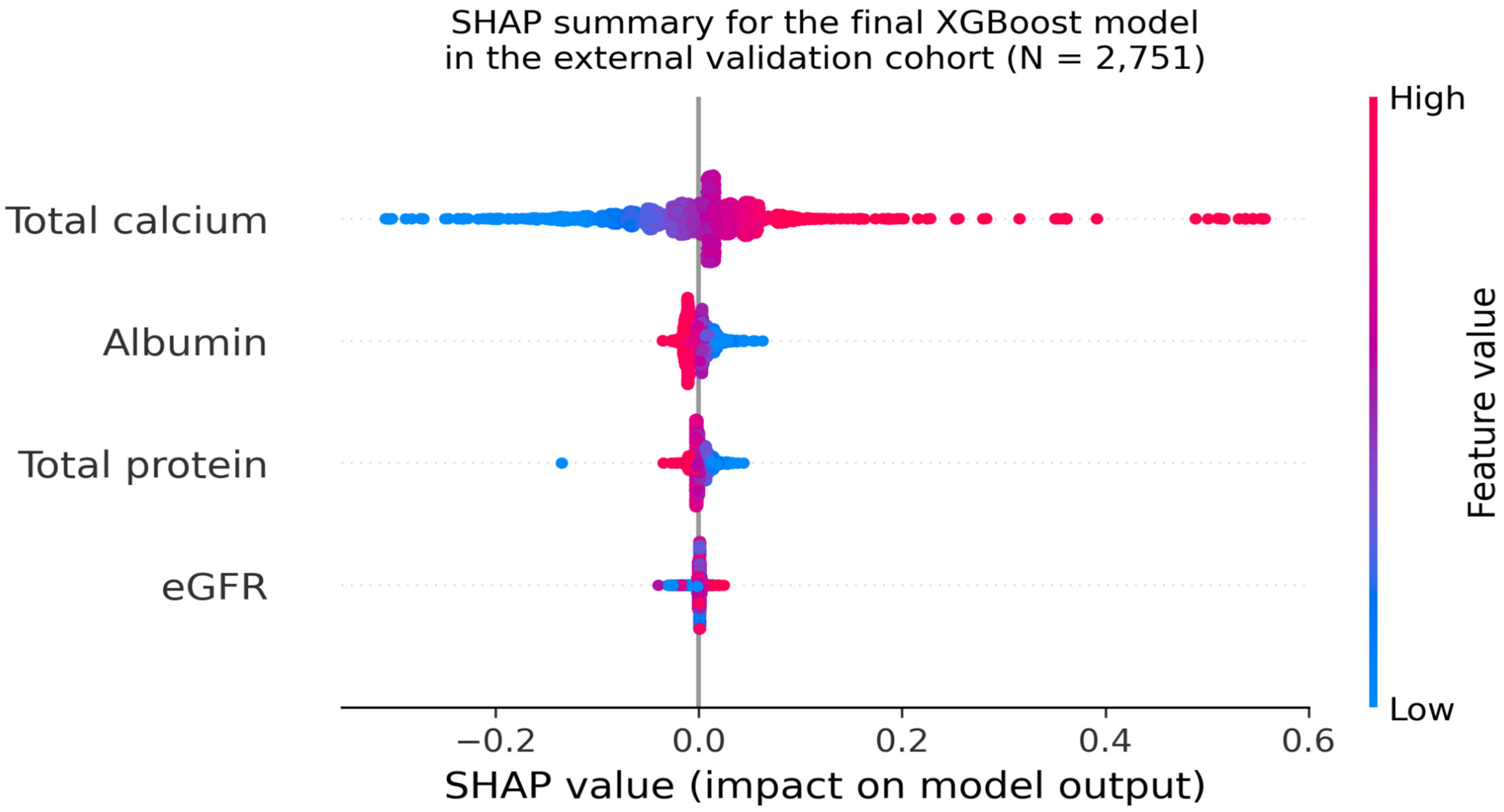

Horizontal SHAP values indicate each feature's contribution to the predicted ionized calcium value, while color represents the observed feature value. SHAP attributions describe model behavior and should not be interpreted as causal effects.

Figure S3. Three-class confusion-matrix heatmaps for total calcium, OLS, and XGBoost in the external validation cohort

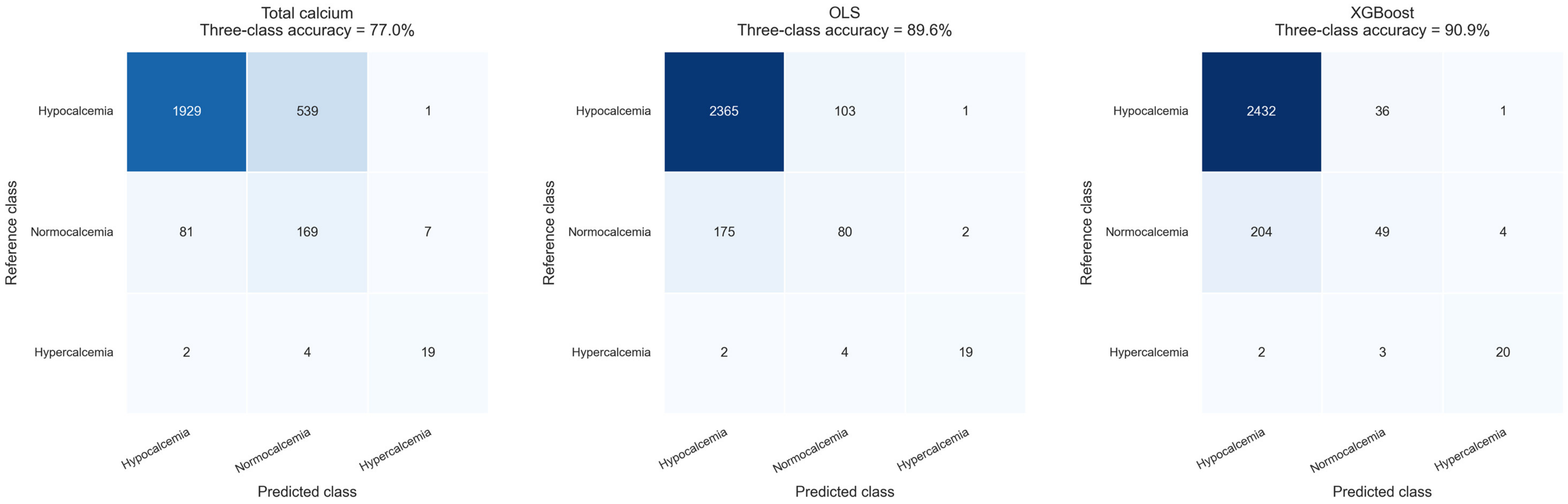

Rows indicate reference classes based on measured ionized calcium and columns indicate predicted classes.

Figure S4. Decision-curve analysis for a hypothetical confirmatory ionized-calcium testing strategy in the external cohort.

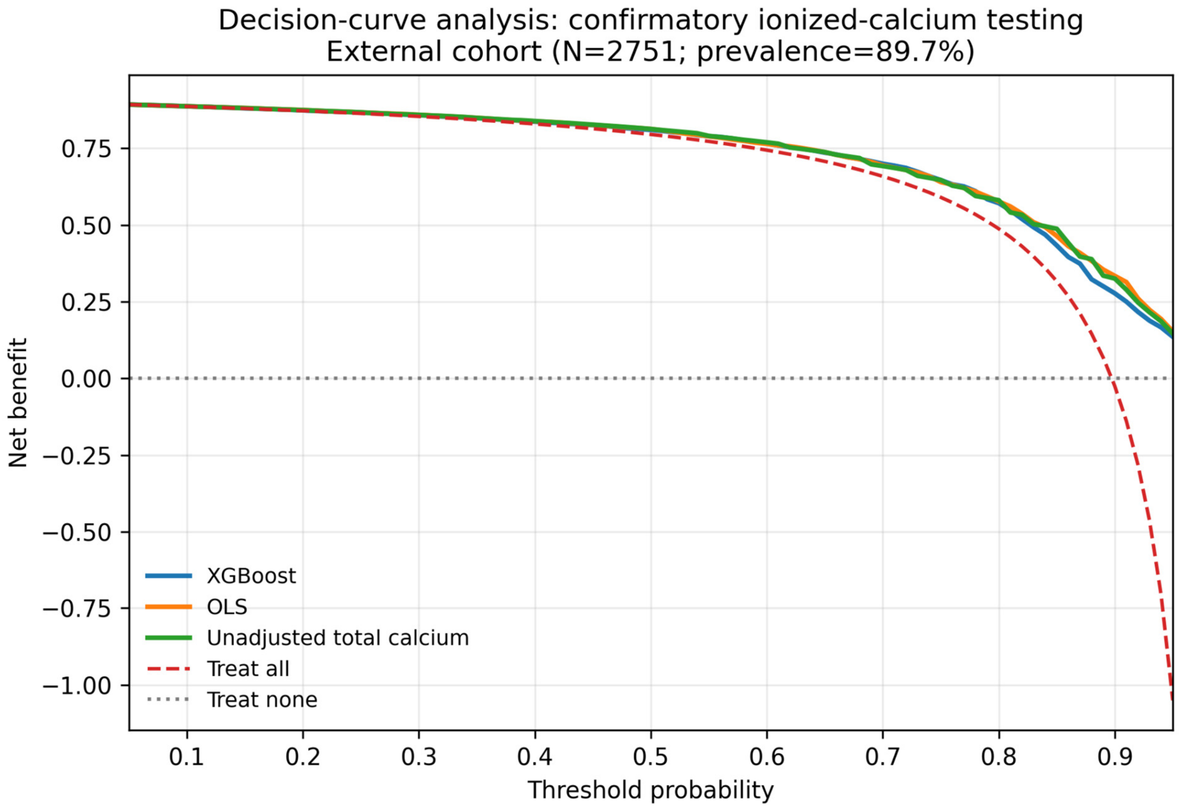

XGBoost, OLS, unadjusted total calcium, treat-all, and treat-none are shown across threshold probabilities 0.05–0.95. Probability mappings were derived in development data and frozen before external application.

Supplementary Tables

Table S1. Adjusted total-calcium formulas and ionized-calcium–estimating equations evaluated in this study

**Formula**	**Equation**	**Output scale**
Simplified Payne	Total calcium (mmol/L) + 0.02 × [40 − Albumin (g/L)]	Adjusted total calcium (mmol/L)
Payne	Total calcium (mg/dL) + 4 − Albumin (g/dL)	Adjusted total calcium (mmol/L)
Orrell	Total calcium (mmol/L) + 0.0177 × [34 − Albumin (g/L)]	Adjusted total calcium (mmol/L)
Berry	Total calcium (mmol/L) + 0.0225 × [46 − Albumin (g/L)]	Adjusted total calcium (mmol/L)
Thode	Total calcium (mmol/L) × [2.7 / (1.7 + Albumin (g/L) / 42)]	Adjusted total calcium (mmol/L)
James	Total calcium (mmol/L) + 0.012 × [39.9 − Albumin (g/L)]	Adjusted total calcium (mmol/L)
Antonio (A)	0.815 × Total calcium $(mmol/L)^{0.5}$	Estimated ionized calcium (mmol/L)
Antonio (B)	0.826 × Total calcium $(mmol/L)^{0.5}$ − 0.023 × renal function (RF);<br>RF=0 if eGFR>60<br>RF=1 if eGFR 30-59<br>RF=2 if eGFR<30	Estimated ionized calcium (mmol/L)
Antonio (C)	0.813 × Total calcium $(mmol/L)^{0.5}$-0.006 × $Albumin^{0.75}$(g/L) + 0.079	Estimated ionized calcium (mmol/L)
Pekar	2.567-2.045e^{-3} × Albumin (g/L) − 5.601e^{-4} × Creatinine (mg/L) + 0.4493 × Total calcium (mmol/L) − 0.307 × pH	Estimated ionized calcium (mmol/L)
Jain	Total calcium (mmol/L) + 0.01 × [30- Albumin (g/L)]	Adjusted total calcium (mmol/L)
Dent	Total calcium (mg/dL) − 0.675 × [Total protein (g/dL) −7.2]	Adjusted total calcium (mmol/L)
Parfitt	Total calcium (mmol/L) / [0.55 + (Total protein (g/L) / 160)]	Adjusted total calcium (mmol/L)
Walker	Total calcium (mg/dL) + 0.92 × [4 − Albumin (g/dL)]	Adjusted total calcium (mmol/L)
Clase	Total calcium (mmol/L) + 0.018 × [35 − Albumin (g/L)]	Adjusted total calcium (mmol/L)
Ridefelt	Total calcium (mmol/L) − 0.0135 × Albumin (g/L) + 0.4525	Adjusted total calcium (mmol/L)

Outputs expressed on the adjusted total-calcium scale were numerically aligned to the ionized-calcium scale using the transformation described in the main Methods; equations directly estimating ionized calcium were retained on their native scale. Measured ionized calcium was the common reference outcome. This alignment was used solely for comparative evaluation and does not represent a physiological conversion between total and ionized calcium. Albumin and total-protein units are specified within each equation. RF denotes the ordinal renal-function category used in the Antonio (B) equation.

Table S2. Frequency of complete XGBoost hyperparameter combinations selected across the 10 outer nested cross-validation folds

n_estimators	max_depth	learning_rate	subsample	colsample_bytree	Outer folds, n	Frequency, %	Final model
100	4	0.05	0.8	1.0	3	30	Yes
100	3	0.1	1.0	0.8	1	10	No
100	3	0.1	0.8	0.8	1	10	No
200	4	0.05	1.0	0.8	1	10	No
300	3	0.05	0.8	0.8	1	10	No
300	3	0.05	1.0	0.8	1	10	No
200	3	0.05	0.8	0.8	1	10	No
200	4	0.05	1.0	1.0	1	10	No

Notes: One best complete combination was recorded per outer fold (n = 10); eight distinct combinations were observed. The selected combination was the sole most frequent complete combination (3/10); all other combinations appeared in one-fold each. Final model settings also included objective = reg:squarederror and random_state = 42. The model was fitted on the full development cohort with the documented development-derived preprocessing and applied to the external cohort without retuning. Frequencies do not establish statistical optimality.

Table S3. Binary hypocalcemia classification metrics accounting for class imbalance in development and external validation cohorts

Method	TP	FP	FN	TN	Binary accuracy, %	PPV, %	F1, %	Balanced accuracy, %	MCC
**Development cohort (n = 14,745)**									
Simplified Payne	3,490	138	8,681	2,436	40.2	96.2	44.2	61.7	0.205
Total calcium	9,834	1,010	2,337	1,564	77.3	90.7	85.5	70.8	0.358
Jain formula	10,066	1,048	2,105	1,526	78.6	90.6	86.5	71.0	0.370
XGBoost (OOF)	11,989	1,906	182	668	85.8	86.3	92.0	62.2	0.398
**External validation cohort (n = 2,751)**									
Total calcium	1,929	83	540	199	77.4	95.9	86.1	74.3	0.333
OLS	2,365	177	104	105	89.8	93.0	94.4	66.5	0.378
XGBoost	2,432	206	37	76	91.2	92.2	95.2	62.7	0.389

TP, true positive; FP, false positive; FN, false negative; TN, true negative; PPV, positive predictive value; F1, harmonic mean of precision and recall; MCC, Matthews correlation coefficient; OOF, out-of-fold. Values were derived from the original fixed-threshold confusion matrices reported in main Tables 5 and 9. The internal XGBoost values are based on nested cross-validation OOF predictions; external XGBoost and OLS results use the frozen development models. The high hypocalcemia prevalence (82.5% and 89.7%) means binary accuracy, PPV, and F1 should not be interpreted independently of the class-specific results. Balanced accuracy weights sensitivity and specificity equally; MCC summarizes all four confusion-matrix cells.

Table S4. Development-cohort out-of-fold prediction performance across pH categories (n = 14,741)

Panel A1. Regression performance of the original and pH-extended XGBoost models

pH group	N	Model	R^2 (95% CI)	MAE (95% CI)	RMSE (95% CI)	Bias (95% CI)
P1	3130	Model A	0.497 (0.456 to 0.536)	0.0685 (0.0663 to 0.0707)	0.0928 (0.0891 to 0.0964)	-0.0187 (-0.0220 to -0.0156)
P1	3130	Model B	0.518 (0.479 to 0.556)	0.0659 (0.0636 to 0.0682)	0.0909 (0.0870 to 0.0946)	-0.0014 (-0.0046 to 0.0017)
P2	7727	Model A	0.515 (0.480 to 0.547)	0.0608 (0.0594 to 0.0622)	0.0872 (0.0838 to 0.0909)	0.0002 (-0.0017 to 0.0021)
P2	7727	Model B	0.518 (0.483 to 0.549)	0.0603 (0.0589 to 0.0617)	0.0870 (0.0836 to 0.0907)	-0.0007 (-0.0026 to 0.0012)
P3	3884	Model A	0.482 (0.436 to 0.518)	0.0738 (0.0718 to 0.0759)	0.1015 (0.0981 to 0.1049)	0.0128 (0.0097 to 0.0161)
P3	3884	Model B	0.494 (0.451 to 0.529)	0.0714 (0.0694 to 0.0735)	0.1003 (0.0969 to 0.1037)	0.0007 (-0.0024 to 0.0039)

Panel A2. Paired differences (pH-extended model B minus original model A)

pH group	N	ΔR^2 (95% CI)	ΔMAE (95% CI)	ΔRMSE (95% CI)
P1	3130	+0.02084 (+0.01153 to +0.03044)	-0.00251 (-0.00323 to -0.00181)	-0.00194 (-0.00281 to -0.00109)
P2	7727	+0.00262 (+0.00020 to +0.00522)	-0.00047 (-0.00066 to -0.00026)	-0.00024 (-0.00047 to -0.00002)
P3	3884	+0.01221 (+0.00457 to +0.02039)	-0.00237 (-0.00290 to -0.00183)	-0.00120 (-0.00197 to -0.00045)

Notes: P1, acidemia (pH < 7.35); P2, normal pH (7.35–7.45, inclusive); P3, alkalemia (pH > 7.45). Model A, original XGBoost model using total calcium, albumin, total protein, and eGFR; Model B, pH-extended XGBoost model including the same four predictors plus pH. OOF, out-of-fold; CI, confidence interval; MAE, mean absolute error; RMSE, root mean squared error. MAE, RMSE, and bias are expressed in mmol/L. Δ denotes the paired difference calculated as Model B minus Model A.

Panel B. Hypocalcemia classification at predicted ionized calcium <1.15 mmol/L

pH group	Model	Hypocalcemic / non-hypocalcemic	TP/FP/FN/TN	Sensitivity (95% CI)	Specificity (95% CI)	PPV	NPV	Balanced accuracy	MCC	AUC (95% CI)
P1	Model A	2504 / 626	2477/512/27/114	98.9% (98.4–99.3%)	18.2% (15.4–21.4%)	82.9%	80.9%	58.6%	0.330	0.813 (0.795–0.832)
P1	Model B	2504 / 626	2446/453/58/173	97.7% (97.0–98.2%)	27.6% (24.3–31.3%)	84.4%	74.9%	62.7%	0.387	0.819 (0.801–0.837)
P2	Model A	6485 / 1242	6388/908/97/334	98.5% (98.2–98.8%)	26.9% (24.5–29.4%)	87.6%	77.5%	62.7%	0.406	0.816 (0.804–0.829)
P2	Model B	6485 / 1242	6391/908/94/334	98.6% (98.2–98.8%)	26.9% (24.5–29.4%)	87.6%	78.0%	62.7%	0.409	0.818 (0.805–0.831)
P3	Model A	3178 / 706	3123/489/55/217	98.3% (97.8–98.7%)	30.7% (27.4–34.2%)	86.5%	79.8%	64.5%	0.438	0.769 (0.749–0.789)
P3	Model B	3178 / 706	3143/510/35/196	98.9% (98.5–99.2%)	27.8% (24.6–31.2%)	86.0%	84.8%	63.3%	0.435	0.757 (0.736–0.778)

Notes: Model A = total calcium, albumin, total protein, and eGFR; Model B = A + pH. P1/P2/P3: n = 3,130/7,727/3,884; non-hypocalcemic n = 626/1,242/706. MAE, RMSE, and bias are in mmol/L; bias = predicted minus measured iCa; Δ = B − A. All intervals are 95%. Classification counts are TP/FP/FN/TN. No formal pH-group interaction test or external validation was performed (external pH unavailable).

Table S5. Out-of-fold regression performance of XGBoost predictor-ablation models in the development cohort (n = 14,745)

Model	Predictors	R^2 (95% CI)	MAE, mmol/L (95% CI)	RMSE, mmol/L (95% CI)	Mean bias, mmol/L
A	TC	0.483 (0.460–0.505)	0.0676 (0.0667–0.0687)	0.0940 (0.0920–0.0961)	-0.00054
B	TC + albumin	0.495 (0.472–0.517)	0.0664 (0.0654–0.0674)	0.0929 (0.0909–0.0950)	-0.00056
C	TC + albumin + total protein	0.497 (0.473–0.518)	0.0662 (0.0652–0.0672)	0.0928 (0.0908–0.0949)	-0.00052
D	TC + albumin + total protein + eGFR	0.500 (0.477–0.522)	0.0659 (0.0649–0.0669)	0.0924 (0.0904–0.0946)	-0.00052

All four models used identical patients and outer cross-validation folds. CIs are patient-level nonparametric bootstrap percentile intervals (2,000 resamples). Model D reproduces the original four-variable model within rounding.

Table S6. Paired differences in out-of-fold performance between predictor-ablation models

Comparison	ΔR^2 (95% CI)	ΔMAE, mmol/L (95% CI)	ΔRMSE, mmol/L (95% CI)
B − A (albumin added)	+0.0122 (+0.0087 to +0.0156)	-0.00125 (-0.00153 to -0.00099)	-0.00112 (-0.00142 to -0.00080)
C − A	+0.0133 (+0.0093 to +0.0171)	-0.00141 (-0.00172 to -0.00112)	-0.00122 (-0.00157 to -0.00085)
D − A (full vs. TC-only)	+0.0169 (+0.0128 to +0.0210)	-0.00173 (-0.00205 to -0.00143)	-0.00155 (-0.00192 to -0.00118)
C − B (total protein added)	+0.0011 (-0.0009 to +0.0031)	-0.00017 (-0.00033 to -0.00000)	-0.00010 (-0.00028 to +0.00008)
D − C (eGFR added)	+0.0036 (+0.0015 to +0.0059)	-0.00032 (-0.00047 to -0.00017)	-0.00034 (-0.00054 to -0.00014)

Table S7. Hypocalcemia classification by the four XGBoost ablation models at the fixed ionized-calcium threshold of <1.15 mmol/L (development cohort; n = 14,745)

Model	TP	FP	FN	TN	Sensitivity, %	Specificity, %	PPV, %	NPV, %	ROC-AUC
A	11973	1955	198	619	98.4	24.0	86.0	75.8	0.803
B	11972	1906	199	668	98.4	26.0	86.3	77.0	0.802
C	11985	1905	186	669	98.5	26.0	86.3	78.2	0.798
D	11989	1906	182	668	98.5	26.0	86.3	78.6	0.798

TP, true positive; FP, false positive; FN, false negative; TN, true negative; PPV, positive predictive value; NPV, negative predictive value. Compared with Model A, Model D had 16 fewer false negatives and 49 fewer false positives, but its ROC-AUC was 0.798 versus 0.803 for Model A. These internal results do not establish clinical benefit or external generalizability.

Table S8. External ROC-AUC estimates and paired DeLong comparisons (n = 2751)

Panel A. Individual ROC-AUC estimates and DeLong 95% confidence intervals

Method	ROC-AUC	95% CI
XGBoost	0.818	0.792–0.845
OLS	0.823	0.797–0.850
Total calcium	0.819	0.792–0.845

Panel B. Paired ROC-AUC differences and multiplicity-adjusted tests

Comparison	AUC 1	AUC 2	ΔAUC	95% CI for ΔAUC	DeLong p	Holm p
XGBoost − OLS	0.818	0.823	-0.0051	-0.0105 to 0.0003	0.064	0.192
XGBoost − Total calcium	0.818	0.819	-0.0005	-0.0098 to 0.0088	0.923	0.923
OLS − Total calcium	0.823	0.819	0.0046	-0.0058 to 0.0150	0.384	0.768

ΔAUC is AUC 1 minus AUC 2. The 95% intervals for ΔAUC are unadjusted paired DeLong intervals; Holm-adjusted p-values account for the three comparisons. None of the three comparisons was significant after Holm adjustment. This does not establish equivalence. External sensitivity and specificity at the fixed calcium thresholds are separate from ROC discrimination.

Table S9. External subgroup performance according to observed albumin and eGFR in the independent validation cohort (n = 2751)

Panel A1. Albumin subgroups — regression performance (XGBoost and OLS).

Group	N	Hypo n (%)	Method	R^2 (95% CI)	MAE (95% CI)	RMSE (95% CI)	Bias
A1	468	436 (93.2%)	XGBoost	0.417 (0.209–0.568)	0.063 (0.057–0.069)	0.093 (0.081–0.105)	0.022
A1	468	436 (93.2%)	OLS	0.373 (0.178–0.506)	0.065 (0.059–0.071)	0.096 (0.083–0.110)	0.024
A2	836	751 (89.8%)	XGBoost	0.477 (0.358–0.572)	0.059 (0.055–0.063)	0.079 (0.074–0.083)	0.018
A2	836	751 (89.8%)	OLS	0.453 (0.350–0.532)	0.060 (0.056–0.063)	0.080 (0.075–0.086)	0.014
A3	1447	1282 (88.6%)	XGBoost	0.309 (0.211–0.401)	0.057 (0.054–0.060)	0.080 (0.075–0.086)	0.017
A3	1447	1282 (88.6%)	OLS	0.272 (0.173–0.358)	0.059 (0.057–0.062)	0.082 (0.077–0.088)	0.015

Panel A2. Albumin subgroups — fixed-threshold classification and ROC discrimination

Group	Method	Hypocalcemic / non-hypocalcemic	TP/FP/FN/TN	Sensitivity (95% CI)	Specificity (95% CI)	PPV	NPV	Balanced accuracy	MCC	AUC (95% CI)
A1	XGBoost	436 / 32	420/19/16/13	96.3% (94.1–97.7%)	40.6%† (25.5–57.7%)	95.7%	44.8%	68.5%	0.387	0.850 (0.778–0.909)
A1	OLS	436 / 32	416/17/20/15	95.4% (93.0–97.0%)	46.9%† (30.9–63.6%)	96.1%	42.9%	71.1%	0.406	0.856 (0.784–0.914)
A1	Total calcium	436 / 32	414/16/22/16	95.0% (92.5–96.6%)	50.0%† (33.6–66.4%)	96.3%	42.1%	72.5%	0.415	0.856 (0.786–0.915)
A2	XGBoost	751 / 85	738/58/13/27	98.3% (97.1–99.0%)	31.8% (22.8–42.3%)	92.7%	67.5%	65.0%	0.425	0.839 (0.788–0.885)
A2	OLS	751 / 85	720/50/31/35	95.9% (94.2–97.1%)	41.2% (31.3–51.8%)	93.5%	53.0%	68.5%	0.415	0.847 (0.797–0.892)
A2	Total calcium	751 / 85	660/28/91/57	87.9% (85.4–90.0%)	67.1% (56.5–76.1%)	95.9%	38.5%	77.5%	0.435	0.844 (0.793–0.889)
A3	XGBoost	1282 / 165	1274/129/8/36	99.4% (98.8–99.7%)	21.8% (16.2–28.7%)	90.8%	81.8%	60.6%	0.392	0.789 (0.751–0.825)
A3	OLS	1282 / 165	1229/110/53/55	95.9% (94.6–96.8%)	33.3% (26.6–40.8%)	91.8%	50.9%	64.6%	0.353	0.794 (0.758–0.830)
A3	Total calcium	1282 / 165	855/39/427/126	66.7% (64.1–69.2%)	76.4% (69.3–82.2%)	95.6%	22.8%	71.5%	0.282	0.796 (0.759–0.831)

Group definitions: A1, <25 g/L; A2, 25–<30 g/L; A3, 30–<35 g/L. † Specificity is based on only 32 non-hypocalcemic patients in A1; confidence intervals are correspondingly wide.

Panel B1. eGFR subgroups — regression performance (XGBoost and OLS)

Group	N	Hypocalcemic n (%)	Method	R^2 (95% CI)	MAE (95% CI)	RMSE (95% CI)	Bias
E1	464	415 (89.4%)	XGBoost	0.590 (0.473–0.679)	0.062 (0.057–0.067)	0.082 (0.075–0.089)	0.020
E1	464	415 (89.4%)	OLS	0.559 (0.449–0.641)	0.063 (0.058–0.068)	0.085 (0.077–0.092)	0.015
E2	546	481 (88.1%)	XGBoost	0.425 (0.255–0.548)	0.060 (0.056–0.065)	0.083 (0.075–0.092)	0.019
E2	546	481 (88.1%)	OLS	0.389 (0.232–0.508)	0.063 (0.058–0.068)	0.086 (0.078–0.094)	0.015
E3	1741	1573 (90.4%)	XGBoost	0.287 (0.185–0.382)	0.057 (0.055–0.060)	0.082 (0.076–0.088)	0.018
E3	1741	1573 (90.4%)	OLS	0.252 (0.157–0.338)	0.059 (0.056–0.062)	0.084 (0.078–0.090)	0.016

Panel B2. eGFR subgroups — fixed-threshold classification and ROC discrimination

Group	Method	Hypocalcemic / non-hypocalcemic	TP/FP/ FN/TN	Sensitivity (95% CI)	Specificity (95% CI)	PPV	NPV	Balanced accuracy	MCC	AUC (95% CI)
E1	XGBoost	415 / 49	409/33/6/16	98.6% (96.9–99.3%)	32.7%† (21.2–46.6%)	92.5%	72.7%	65.6%	0.451	0.864 (0.811–0.910)
E1	OLS	415 / 49	402/29/13/20	96.9% (94.7–98.2%)	40.8%† (28.2–54.8%)	93.3%	60.6%	68.8%	0.451	0.873 (0.821–0.920)
E1	Total calcium	415 / 49	355/15/60/34	85.5% (81.8–88.6%)	69.4%† (55.5–80.5%)	95.9%	36.2%	77.5%	0.420	0.860 (0.803–0.911)
E2	XGBoost	481 / 65	474/40/7/25	98.5% (97.0–99.3%)	38.5% (27.6–50.6%)	92.2%	78.1%	68.5%	0.510	0.831 (0.775–0.882)
E2	OLS	481 / 65	463/33/18/32	96.3% (94.2–97.6%)	49.2% (37.5–61.1%)	93.3%	64.0%	72.7%	0.511	0.837 (0.782–0.888)
E2	Total calcium	481 / 65	366/21/115/44	76.1% (72.1–79.7%)	67.7% (55.6–77.8%)	94.6%	27.7%	71.9%	0.312	0.835 (0.782–0.884)
E3	XGBoost	1573 / 168	1549/133/24/35	98.5% (97.7–99.0%)	20.8% (15.4–27.6%)	92.1%	59.3%	59.7%	0.315	0.802 (0.763–0.836)
E3	OLS	1573 / 168	1500/115/73/53	95.4% (94.2–96.3%)	31.5% (25.0–38.9%)	92.9%	42.1%	63.5%	0.307	0.804 (0.766–0.838)
E3	Total calcium	1573 / 168	1208/47/365/121	76.8% (74.6–78.8%)	72.0% (64.8–78.3%)	96.3%	24.9%	74.4%	0.321	0.801 (0.764–0.835)

Group definitions: E1, <30; E2, 30–<60; E3, ≥60 mL/min/1.73 m². † Specificity is based on only 49 non-hypocalcemic patients in E1; confidence intervals are correspondingly wide.

Notes: Hypo/non, hypocalcemic/non-hypocalcemic patients; TP, true positive; FP, false positive; FN, false negative; TN, true negative; PPV/NPV, positive/negative predictive values; MCC, Matthews correlation coefficient. Regression error and bias are in mmol/L. Confidence intervals are 95%. No formal subgroup interaction tests were performed. The subgroup confusion matrices sum to the previously reported overall external results for each method.

Table S10. Development-derived winsorization bounds applied to the external validation cohort, with the number of external values below, above, and winsorized at each bound

Predictor	Development lower bound	Development upper bound	External n below	External n above	External n winsorized
tc	1.230	2.924	0	17	17
alb	6.500	52.000	1	0	1
stp	19.900	97.600	4	3	7
eGFR	-170.060	286.340	0	0	0

All bounds were learned exclusively from the development cohort using Q1 − 3×IQR and Q3 + 3×IQR and applied unchanged to the external cohort.

Table S11. SHAP-based feature importance of the final XGBoost model in the external validation cohort (n = 2751)

**Feature**	**Mean ∣SHAP∣**	**Median ∣SHAP∣**	**Relative SHAP share, %**	**Rank**
Total calcium	0.048500	0.033432	74.0	1
Albumin	0.009431	0.009033	14.4	2
Total protein	0.005678	0.003538	8.7	3
eGFR	0.001944	0.001096	3.0	4

Table S12. External threshold-matched comparison of XGBoost and unadjusted total calcium (n = 2751).

Method / threshold	Development matching criterion	External sensitivity, %	External specificity, %	Δ sensitivity vs TC (95% CI)	Δ specificity vs TC (95% CI)
Total calcium <2.1956 mmol/L	Fixed clinical benchmark	78.1	70.6	Reference	Reference
XGBoost <1.15 mmol/L	Original fixed iCa cutoff	98.5	27.0	—	—
XGBoost <1.0872 mmol/L	Match TC development specificity (60.8%)	77.6	70.2	-0.005 (-0.015 to 0.005)	-0.004 (-0.038 to 0.032)
XGBoost <1.0853 mmol/L	Match TC development sensitivity (80.8%)	75.9	73.4	-0.022 (-0.032 to -0.012)	+0.028 (0.000 to 0.062)

Notes: TC, unadjusted total calcium. Positive class: measured ionized calcium <1.15 mmol/L. The matched XGBoost thresholds were selected from development out-of-fold predictions only and were not re-optimized in the external cohort. Therefore, sensitivity or specificity was not expected to remain exactly matched after transport to the external cohort. Δ values are XGBoost minus total calcium. Confidence intervals are paired patient-level bootstrap percentile intervals (2,000 resamples; seed 42). The threshold-matched results do not show a clear incremental external classification advantage for XGBoost over unadjusted total calcium.

Table S13. Decision-curve analysis in the independent external validation cohort (n = 2751).

Panel A. Development-derived probability mappings.

Method	Calibration score	Intercept	Coefficient	Development Brier
XGBoost	-OOF predicted iCa	24.5749	21.3629	0.1087
OLS	-OOF predicted iCa	20.0207	17.0263	0.1093
Unadjusted total calcium	-Observed total calcium	15.1102	6.3065	0.1120

Panel B. External probability calibration (diagnostic only).

Method	Mean predicted probability	Observed prevalence	Brier score	Calibration intercept	Calibration slope
XGBoost	0.8255	0.8975	0.0773	0.5844	1.1439
OLS	0.8326	0.8975	0.0757	0.4458	1.2094
Unadjusted total calcium	0.8370	0.8975	0.0757	0.2744	1.2953

Panel C. External net benefit at selected threshold probabilities.

pt	Treat all	TC	OLS	XGBoost	ΔNB XGB−TC (95% CI)	ΔNB OLS−TC (95% CI)
0.10	0.8861	0.8867	0.8868	0.8867	0.0001 (-0.0008 to 0.0006)	0.0001 (-0.0000 to 0.0003)
0.20	0.8719	0.8741	0.8745	0.8733	-0.0008 (-0.0027 to 0.0008)	0.0004 (-0.0006 to 0.0011)
0.30	0.8536	0.8585	0.8586	0.8575	-0.0010 (-0.0034 to 0.0011)	0.0001 (-0.0017 to 0.0018)
0.40	0.8292	0.8386	0.8379	0.8365	-0.0021 (-0.0056 to 0.0012)	-0.0007 (-0.0035 to 0.0019)
0.50	0.7950	0.8128	0.8128	0.8095	-0.0033 (-0.0087 to 0.0022)	0.0000 (-0.0040 to 0.0040)

Notes: pt, threshold probability; NB, net benefit; TC, unadjusted total calcium. Probability mappings were fitted only in the development cohort and applied unchanged externally. External hypocalcemia prevalence was 89.7%. Across the full 0.05–0.95 grid, mean ΔNB for XGBoost versus TC was -0.0051 (maximum 0.0157). Bootstrap confidence intervals at the prespecified thresholds included zero. XGBoost and OLS exceeded treat-all over 0.05–0.95; TC exceeded treat-all over 0.06–0.95. These findings do not establish XGBoost-specific clinical decision benefit.
